# Clinicopathological features and genetic mutation spectrum of primary anastomosing hemangioma arising from the kidney

**DOI:** 10.3389/fimmu.2025.1554203

**Published:** 2025-05-09

**Authors:** Luxin Zhang, Haozhen Li, Heyao Tong, Hepeng Cui, Huahang Guo, Shuang Wen, Zhuwei Song, Jiaqiang Chen, Shengxiang Xiang, Zhiyu Liu, Bo Fan, Liang Wang

**Affiliations:** ^1^ Department of Urology, Second Affiliated Hospital of Dalian Medical University, Dalian, Liaoning, China; ^2^ Liaoning Provincial Key Laboratory of Urological Digital Precision Diagnosis and Treatment, Dalian, Liaoning, China; ^3^ Liaoning Engineering Research Center of Integrated Precision Diagnosis and Treatment Technology for Urological Cancer, Dalian, Liaoning, China; ^4^ Dalian Key Laboratory of Prostate Cancer Research, Dalian, Liaoning, China; ^5^ Department of Pathology, Dalian Friendship Hospital, Dalian, Liaoning, China

**Keywords:** renal anastomosing hemangiomas, whole-genome sequencing, gene mutations, immunoregulation, population-based study

## Abstract

**Background:**

Renal anastomosing hemangioma (RAH) is a rare benign renal tumor, and its clinicopathologic characteristics and genetic mutation spectrum related to its mechanisms of pathogenesis are unclear.

**Methods:**

We carried out whole-genome sequencing (WGS) on RAH samples to explore the genetic mutation spectrum and verified the results by Sanger sequencing. Immunohistochemical analysis was also performed to reveal the histopathological characteristics and the tumor microenvironment components. Moreover, a population-based study was conducted after searching the PubMed, EMBASE, and Ovid SP databases to systematically summarize the clinicopathologic features of patients with RAH.

**Results:**

WGS analysis revealed 10532 somatic single-nucleotide variants (SNVs), 6705 somatic insertions and deletions (INDELs), and mutations in 32 predisposing genes and 10 driver genes, among which the mutations in 8 of the predisposing genes, *CNTNAP2*, *NCOA2*, *FAT1*, *MET*, *TJP2*, *MAML2*, *SRGAP3*, and *CSMD3*, and the mutation site in the driver gene *HIP1* were confirmed by Sanger sequencing. Moreover, the immunohistochemical profile of the tumor microenvironment revealed that the expression content of tumor-associated macrophages (CD163, CD68) and fibroblasts (SMA) differs between cancerous and precancerous tissues which may regulate the disease development. On the basis of our population-based analysis, we summarized the clinicopathological features of 100 patients with RAH and identified significant differences in age (*p*=0.001), tumor site (*p*<0.001), tumor focality (*p*<0.001), largest tumor diameter (*p*=0.001) and surgical approach (*p*=0.010) between patients with RAH with end-stage renal disease (ESRD) and those without ESRD.

**Conclusions:**

The distinct phenotypes of RAH may be associated with the different genetic mutation spectra identified in our study. The presence or absence of comorbid ESRD varies among patients with RAH. However, additional studies are required to validate our results.

## 1 Introduction

Anastomotic hemangioma (AH) is a rare and histopathologically distinct benign vascular tumor that preferentially involves the genitourinary tract and paraspinal region. In 2009, Montgomery and Epstein first described AH as a distinct vascular lesion in the kidney and testis (1). Renal anastomotic hemangiomas (RAHs) are listed in the 2016 WHO tumor classification as a subtype of renal capillary hemangioma (2). RAH can develop across a broad age spectrum, occurring from 10 to 83 years of age (with an average age of 49 years), with a male-to-female ratio of 2:1 (3). These hemangiomas are often observed in individuals with end-stage renal disease (ESRD), and approximately two-thirds of cases are associated with compromised renal function (4). Although AHs are usually isolated, multifocal and bilateral lesions are also common in patients with ESRD (3, 5).

Most AHs are found incidentally on radiological evaluation for other purposes. Common symptoms of renal hemangiomas include abdominal pain, hematuria, and the presence of an abdominal mass (3, 5). It is challenging to preoperatively diagnose renal hemangiomas by imaging because of their small size, nonspecific imaging characteristics, and difficulty in distinguishing them from other renal tumors (6). Limited information is available regarding the imaging features of renal anastomotic hemangiomas. They typically appear as restricted lesions with high T2 signals on magnetic resonance imaging, peripheral or diffuse enhancement in endoarterial phases on dynamic CT, sustained enhancement in delayed phases, and centripetal filling with strong contrast enhancement from the periphery to the center as potential diagnostic clues (7).

Histologically, RAH tumors consist of splenic sinusoidal vascular channels that are frequently connected and lined with cuboidal endothelial cells (5, 8–12). Endothelial cells usually present as flat nails (9–11). Sometimes, eosinophilic clear spherules are present in the cytoplasm of tumor endothelial cells (9, 11). The stroma is occasionally infiltrated with foamy macrophages or mast cells (11). Extramedullary hematopoiesis is observed in vascular channels containing red lineage precursor cells and megakaryocytes (5, 9, 11, 12). Immunohistochemically, almost all AHs exhibit diffuse positive staining for endothelial markers (including CD31, CD34, ERG, factor VIII, and FLI-1), supportive pericytes prominently express SMA, and lower endothelial cell proliferative activity is suggested given that most of the Ki-67 staining is < 5% (5, 10). The prognoses of patients with AH, and cases of recurrence and death, have not been reported in previous studies, so AH is considered a benign tumor. Since preoperative clinical features are not sufficient for a definitive diagnosis of RAH and almost 90% of patients with renal RAH undergo total nephrectomy, precise preoperative percutaneous puncture is essential to prevent unnecessary surgical procedures (4, 5).

Regarding the genetic features of RAH, it was recently reported that 90% of patients with AH present with the circulating activation of certain hotspot mutations, including *GNAQ*, *GNA14*, or *GNA11*, and that AH is a clonal tumor confirmed by these G proteins. However, these mutated G proteins are expressed in other vascular tumors. Moreover, these mutations are not found in angiosarcomas, which could provide evidence for distinguishing AH from AS (13).

Almost all previous genetic studies of patients with RAH have been based on NGS, and this study is the first in which whole-exome sequencing was performed on a patient with a pathological diagnosis of left renal anastomotic hemangioma, with new findings of somatic mutations, tumor susceptibility genes, and driver genes in several tumor samples. We also conducted a population-based study after searching the relevant literature to summarize the clinical and pathological features, possible prognoses, and treatment strategies for patients with RAH.

## 2 Materials and methods

### 2.1 Clinical analysis

All relevant information was obtained from the Second Hospital of Dalian Medical University. The study protocol received approval from the Ethics Committee (approval number: KY2024-391-01) and adhered to the Helsinki Declaration of Ethical Principles for Human Medical Research. We obtained written informed consent from the patient.

### 2.2 Immunohistochemical analysis

A section of a renal mass was obtained by the laboratory of our hospital. The sample was fixed in 10% formaldehyde, embedded in paraffin, sliced into 4 mm sections, placed on slides, and some of the slices were stained with hematoxylin and eosin (HE) before being photographed under a light microscope. The other slices were stained with various antibodies to stain the markers for diagnosis including CD31, CD34, podoplanin (D2-40), EMA, cytokeratin (AE1/AE2), melanoma gp100 (HMB45), Ki67, FLI1, ERG, GLUT-1, and herpesvirus 8(HHV8) and the markers to detect components of the tumor microenvironment including PD-L1, markers of tumor-associated fibroblasts (SMA), markers of TAMs (Tumor-associated macrophages, CD163 and CD68). The detailed information of antibodies was presented in the **Supplementary Table S1**.

### 2.3 Whole-genome sequencing

The data presented in the study are uploaded in the NCBI database SRA repository under accession numbers SRR28364768 and SRR28364767 (http://www.ncbi.nlm.nih.gov/sra/?term=SRR28364768, https://www.ncbi.nlm.nih.gov/sra/?term=SRR28364767).

#### 2.3.1 DNA extraction

Following the manufacturer’s instructions, DNA was extracted from the FFPE tumor sample and matched peripheral blood sample via the GeneRead DNA FFPE Kit (Qiagen, Germany). Whole-genome sequencing (WGS) was performed on the samples. Using agarose gel electrophoresis and the Qubit^®^ DNA Assay Kit in a Qubit^®^ 3.0 Fluorometer (Invitrogen, USA), DNA quality was assessed. To construct libraries, DNA samples totaling 0.2 µg and with concentrations greater than 20 ng/µL were utilized.

#### 2.3.2 Library preparation and sequencing

Sequencing libraries were created using the NEBNext^®^ UltraTM DNA Library Preparation Kit (NEB, USA), and each sample was given an index code. After the genomic DNA samples were divided into 350 bp fragments using Covaris sonication, the DNA fragments were polished, A-tailed, and ligated to full-length adapters. This was completed before additional PCR amplification. The AMPure XP system (Beckman Coulter, Beverly, USA) was used to purify the PCR products. A Qubit^®^ 3.0 Fluorometer (Invitrogen, USA) was then used to measure the quantities of DNA, and an NGS3K/Caliper was used to analyze the size distribution of the libraries and quantify them using real-time PCR (3 nM). The index-coded samples were clustered using the cBot Cluster Generation System with the Illumina PE150 Cluster Kit (Illumina, USA).

#### 2.3.3 Quality control

The raw data were filtered to remove reads with adapters, more than 10% unidentifiable base information in single-end sequencing reads, and low-quality bases (below the 5th level) accounting for more than 50% of the read length. Reads that were more than half of the length were eliminated. The quality of the sequencing data was ensured to be primarily distributed at a Q30 of not less than 80%, and the sequencing error rate for each base location was less than 1%.

#### 2.3.4 Bioinformatics analysis

To produce the BAM format for the first alignment results, validated sequencing data were aligned using BWA (14) against the reference genome (GRCh37/hg19/GRCh38). After that, SAMtools (14) was used to score the results, and Sambamba was used to identify duplicate reads. Next, Sambamba was used to identify duplicate reads. Ultimately, the depth and coverage statistics of the identified duplicate reads were computed.

#### 2.3.5 Variant detection, somatic mutation calling and functional annotation

We identified somatic variants, including SNPs, INDELs, copy number variants (CNVs) and structural variants (SVs). SAMtools (15) was used to identify SNPs, count the number of SNPs in different genome regions, determine the number of different types of SNPs in coding regions, determine the distribution of conversions and reversals, determine the number of SNPs, and determine the genotype distribution on the basis of the initial results (BAM files). INDELs were identified using SAMtools, which calculates the number of INDELs in different genomic areas and the variety of INDELs in coding regions. The variety of CNV occurrences was determined, and Control-FREEC (16) was utilized to detect increases and decreases in CNVs. Lumpy (17) was used to identify SVs and determine the variety of SV events that could occur. Somatic SNVs, INDELs, CNVs, and SVs were detected using Mutect (18), Strelka (19), Control-FREEC (16), and Lumpy (17). ANNOVAR was used for functional annotation of the identified gene variations (20).

#### 2.3.6 Identification of potential predisposing genes and driver mutations

Using the CGC database (Cancer Gene Census, http://cancer.sanger.ac.uk/cancergenome/projects/census/), FACD (Familial Cancer Database, http://www.familialcancerdatabase.nl/), intOGen (https://intogen.org/search) database, and the reported genes summarized in the Nature literature, we used SAMtools software to detect germline mutations (SNPs, INDELs) in patient normal tissues. Using muTect software, we analyzed somatic mutations (SNVs and INDELs) in tumor tissues. We subsequently searched the CGC database, Bert Vogelstein’s significant mutated genes (SMGs) (21), synthesis (22), and known driver genes.

#### 2.3.7 Analysis of tumor purity, tumor ploidy and targeted drug prediction

We calculated the purity of the tumor sample (the ratio of tumor cells to total cells), ploidy (the average copy number of the sample), and cancer DNA fraction (the ratio of tumor DNA to total DNA) using ABSOLUTE to guarantee the quality of the analyses (23) We compared the identified somatic mutations with NovoDR medication databases, such as the My Cancer Genome, FDA, and Pharmacogenomics Knowledge Base Database (PharmGKB) (24), to evaluate potential targeted drugs.

#### 2.3.8 Sanger sequencing

Sanger sequencing was used to validate the susceptibility and driver genes containing mutant bases identified by NGS. Following the manufacturer’s instructions, genomic DNA was extracted, and polymerase chain reaction (PCR) was carried out. Once the PCR products were purified via agarose electrophoresis, an Applied BiosystemsTM 3730xl sequencer was used to sequence the products.

#### 2.3.9 Structure of the protein–protein interaction network of mutant genes

To construct the protein–protein interaction (PPI) network, we searched the genes confirmed by Sanger sequencing with the Search Tool for the Retrieval of Interacting Genes/Proteins (STRING, version 12.0) database, set the minimum required interaction score at 0.40 and the maximum number of interactors at 50, and then visualized the PPI network with Cytoscape software (version 3.9.1).

#### 2.3.10 GO and KEGG pathway enrichment analyses

We analyzed the biological functions of the PPI node genes through Gene Ontology (GO) (25)and Kyoto Encyclopedia of Genes and Genomes (KEGG) (26) pathway enrichment analyses with a statistically significant difference threshold of q value < 0.05 and visualized the data in plots constructed via R software (version 4.3.2).

#### 2.3.11 Relationship between predisposing gene mutations and immune cell infiltration in the tumor microenvironment

We searched the mutated genes and surface antigens of immune cells in the tumor microenvironment and analyzed the combined score with the STRING database, setting the minimum required interaction score at 0.15, and then visualized the PPI network with Cytoscape software (version 3.9.1).

### 2.4 Population-based study

We searched the relevant literature in the PubMed, EMBASE, and Ovid SP databases for RAH patients before February 2025 using the keywords “kidney”, “renal” and “anastomosing hemangioma”. Only patients for whom complete information was available were included in our analysis. A total of 100 patients were included, as shown in the detailed flow chart in **Supplementary Figure S1**. We summarized their clinical characteristics, including age, sex, clinical manifestations, clinical history of end-stage renal disease (ESRD), preoperative diagnosis and surgical approach; pathological features, including tumor site and location; tumor focality; largest tumor diameter (mm); concurrent or history of other renal tumors; and immunohistochemistry results. We divided the patients into an ESRD group and a non-ESRD group, compared the continuous variables using Student’s t test or the Mann–Whitney U test, and analyzed the classification indicators by the chi-square test or Fisher’s exact test. The significance level was established as *p* < 0.05, and all the statistical analyses were performed using SPSS version 26.0 (SPSS Inc., Chicago, IL, USA). Moreover, we searched the SEER database (https://seer.cancer.gov/), which consists of 17 registries covering more than 26.5% of the United States population between 2000 and 2021, for information on hemangiosarcoma patients. Patients with an International Classification of Disease (ICD)-O-3 code of 9120 with malignant behavior were screened. We included a total of 53 renal hemangiosarcoma patients in our study after excluding patients lacking histological confirmation or with unknown survival times. The clinical and tumor characteristics, including age, sex, tumor site and tumor size, were obtained, and survival outcomes, including overall survival (OS) and cancer-specific survival (CSS), were also downloaded. We compared the continuous variables between RAH patients and renal hemangiosarcoma patients using Student’s t test or the Mann–Whitney U test and analyzed the classification indicators between RAH patients and renal hemangiosarcoma patients via the chi-square test or Fisher’s exact test. Survival analysis was performed using GraphPad.

## 3 Results

### 3.1 Clinical analysis

#### 3.1.1 Case presentation

**Figure 1:**
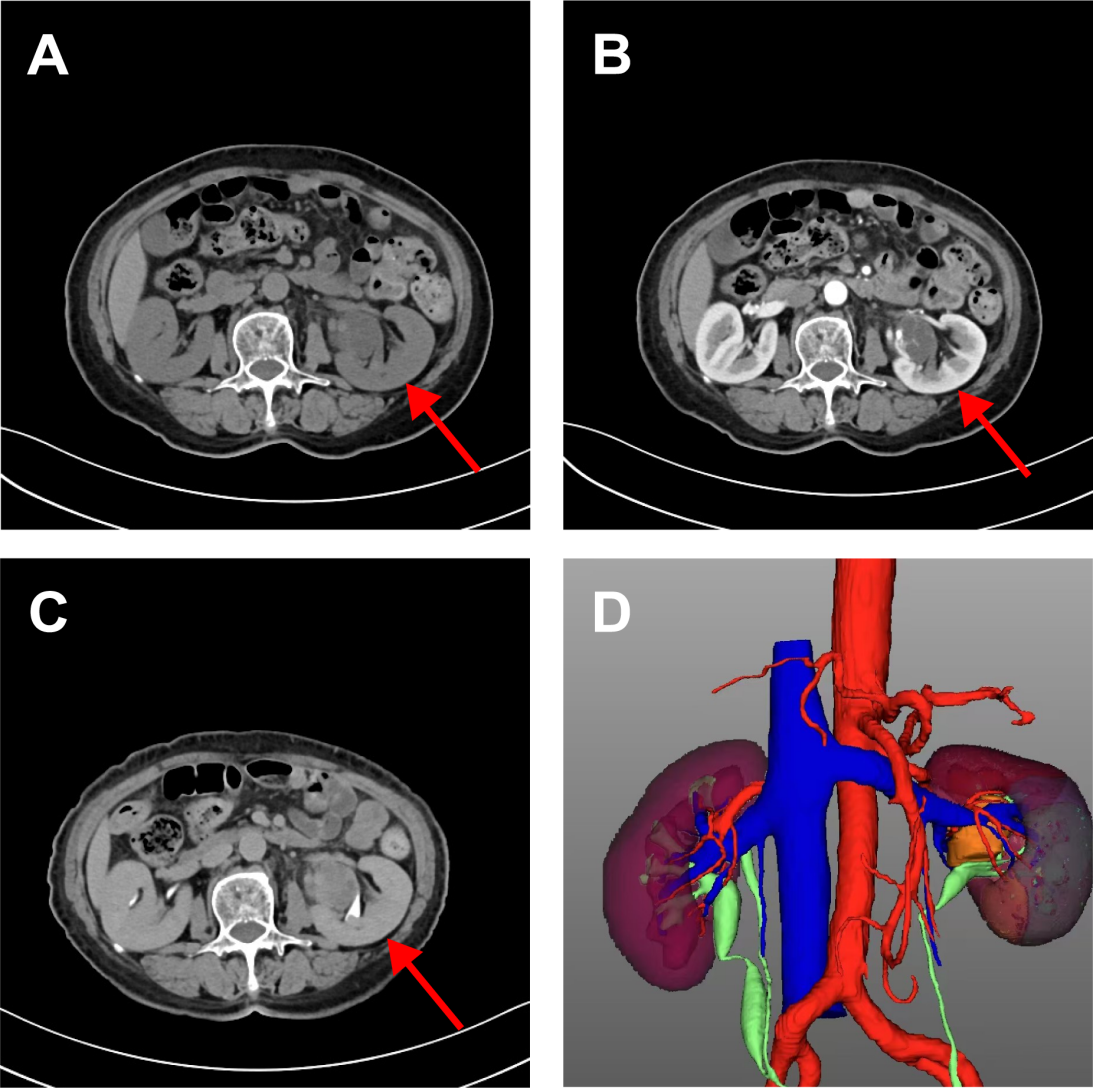
CT urography imaging of renal anastomosing hemangioma. Flat sweep **(A)** enhanced cortical phase; **(B)** enhancing draining phase; **(C)** and reconstructed three-dimensional images **(D)**. It showed a cystic solid space-occupying lesion adjacent to the left renal pelvis measuring 3.4 cm*2.6 cm. It is poorly demarcated from the adjacent ureter and is considered a possible low-grade malignant neoplastic lesion. The reconstructed three-dimensional clearly showed the lesion and surrounding anatomical structure.

A 71-year-old woman with a left renal pelvic mass was identified via physical examination at an outside hospital. Her medical records revealed type 2 diabetes, hypertension, and coronary artery disease. She refuted having any other medical history, including end-stage renal disease (ESRD). The patient had no symptoms of left-sided low back pain, hematuria to the naked eye, or an abdominal mass. Preoperatively, the patient’s urine was retained for fluid-based cytology, suggesting that no uroepithelial cells were present. Preoperative urologic ultrasound revealed a mixed mass in the left renal pelvis measuring approximately 4.0*3.3 cm with clear borders and regular morphology, which was suspected to be cystic nephrocalcinosis of the left kidney. To further clarify the nature of the left renal pelvis tumor, CTU examination revealed a cystic solid-occupying lesion in the left pararenal pelvis measuring 3.4 cm*2.6 cm. Enhanced scanning revealed obvious enhancement of the solid portion, with persistent enhancement in the delayed phase and unclear demarcation from the adjacent ureter, which was considered to indicate a possible low-grade malignant neoplastic lesion, as shown in **Figure 1**, and the lesion and surrounding anatomical structure were clearly visible in the reconstructed three-dimensional images. Owing to the clinical diagnosis of a left renal pelvic mass, malignancy was not excluded, so the patient underwent robotic-assisted laparoscopic partial left nephrectomy under general anesthesia. Postoperative pathology suggested renal anastomotic hemangioma. Regular postoperative telephone follow-ups were conducted for 27 months, with the last follow-up occurring in February 2025, and no tumor recurrence or metastasis was observed.

#### 3.1.2 Histopathological considerations

The size of the macroscopic tissue sample of the left pararenal mass was 2.4 cm * 2.2 cm * 1. The mass section was cystic and soft, had a mass adjacent to the peeling edge, did not show obvious necrosis, and contained a large amount of free adipose tissue. The size of the mass was 5×4.5×1.5 cm. The grayish-yellow texture was soft, and the mass could be a palpable nodule. Microscopic observation (left renal tumor base): tumor cells are polygonal or ovoid, with clear borders, clustered or scattered distribution, and proliferation of interstitial fibrous tissue, blood vessels, and adipose tissue. A benign or low-grade malignant tumor was considered. Short spindle-shaped, lattice-like, slit-like, irregularly dilated large blood vessels were observed, acute inflammatory cells and plasma cells were absent, and adipose tissue was also observed. No fibrin-like microthrombi, extramedullary hematopoiesis or lymphocytic infiltration was observed in focal areas within the tumor. No definitive diagnosis was made, and a benign or low-grade malignant tumor was initially considered, with immunohistochemistry required for further diagnosis.

#### 3.1.3 Immunohistochemical profile for diagnosis

**Figure 2:**
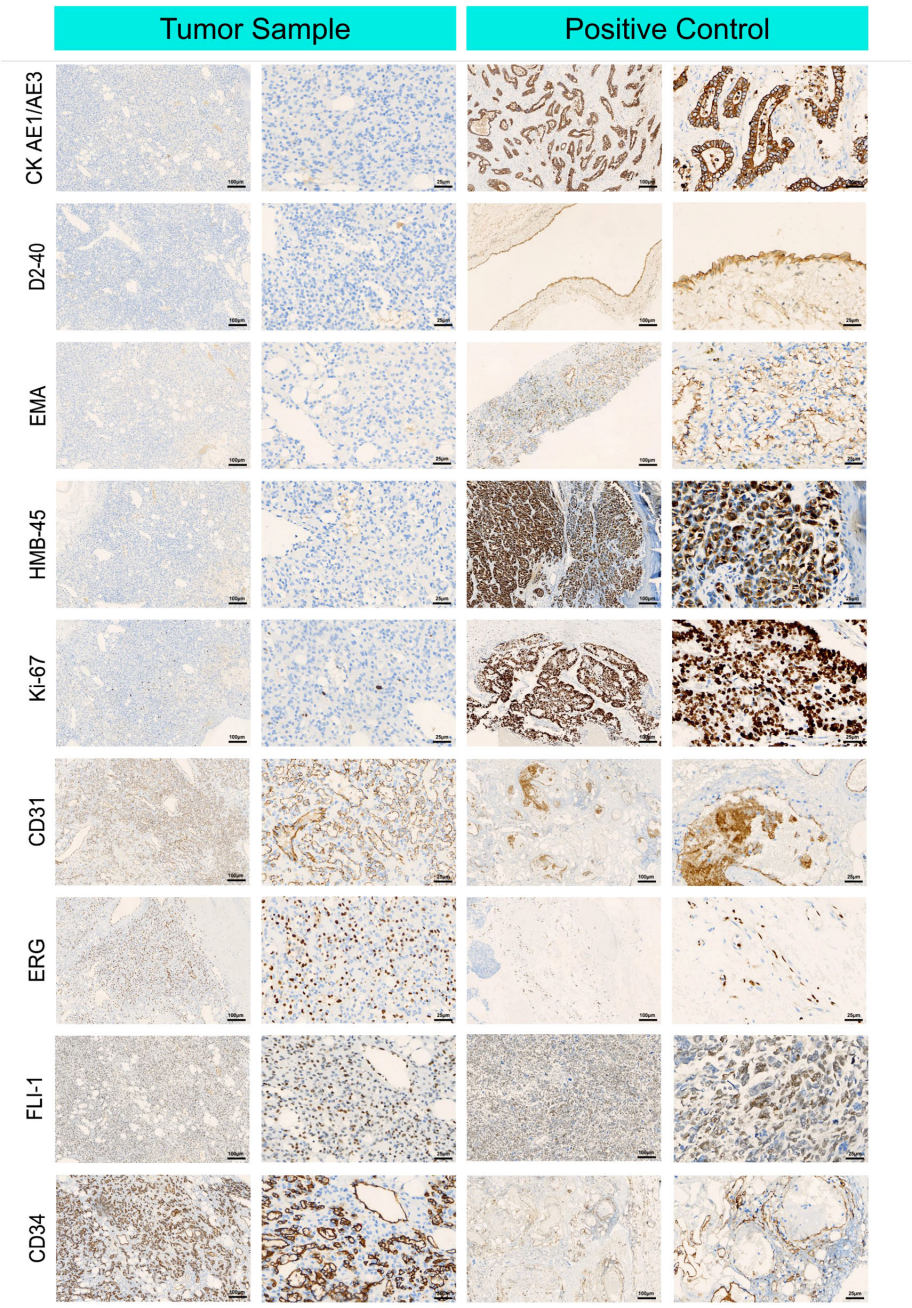
Immunohistochemical microscopic images of renal anastomosing hemangioma tumor cells. Representative immunohistochemical images of cluster of differentiation (CD) 31, cluster of differentiation (CD) 34, cytokeratin AE1/AE 3, lymphatic vessel marker D2-40, epithelial membrane antigen EMA, endothelial cell markers ERG, FLI-1, glucose transporter GLUT-1, glycoprotein HMB-45, Ki-67, and smooth muscle actin SMA, which were in tumor cells were positive in tumor cells expressing endothelial cell markers.

**Figure 2** shows diffuse staining for endothelial markers via tumor immunohistochemistry. The endothelial cell markers CD31, CD34, ERG, and FLI-1 were diffusely expressed in the AH cells. Negative expression of D2–40 differentiated the tumor from lymphoid-derived tumors. GLUT-1 negativity excluded juvenile hemangiosarcoma. Negative HMB-45 differentiated the tumor from Kaposi’s sarcoma. Tumors expressing SMA and Ki-67 markers can be distinguished from hemangiosarcoma, which has a relatively high Ki-67 proliferation index but no pericyte SMA expression.

#### 3.1.4 Immunohistochemistry for detecting components of the tumor microenvironment

**Figure 3:**
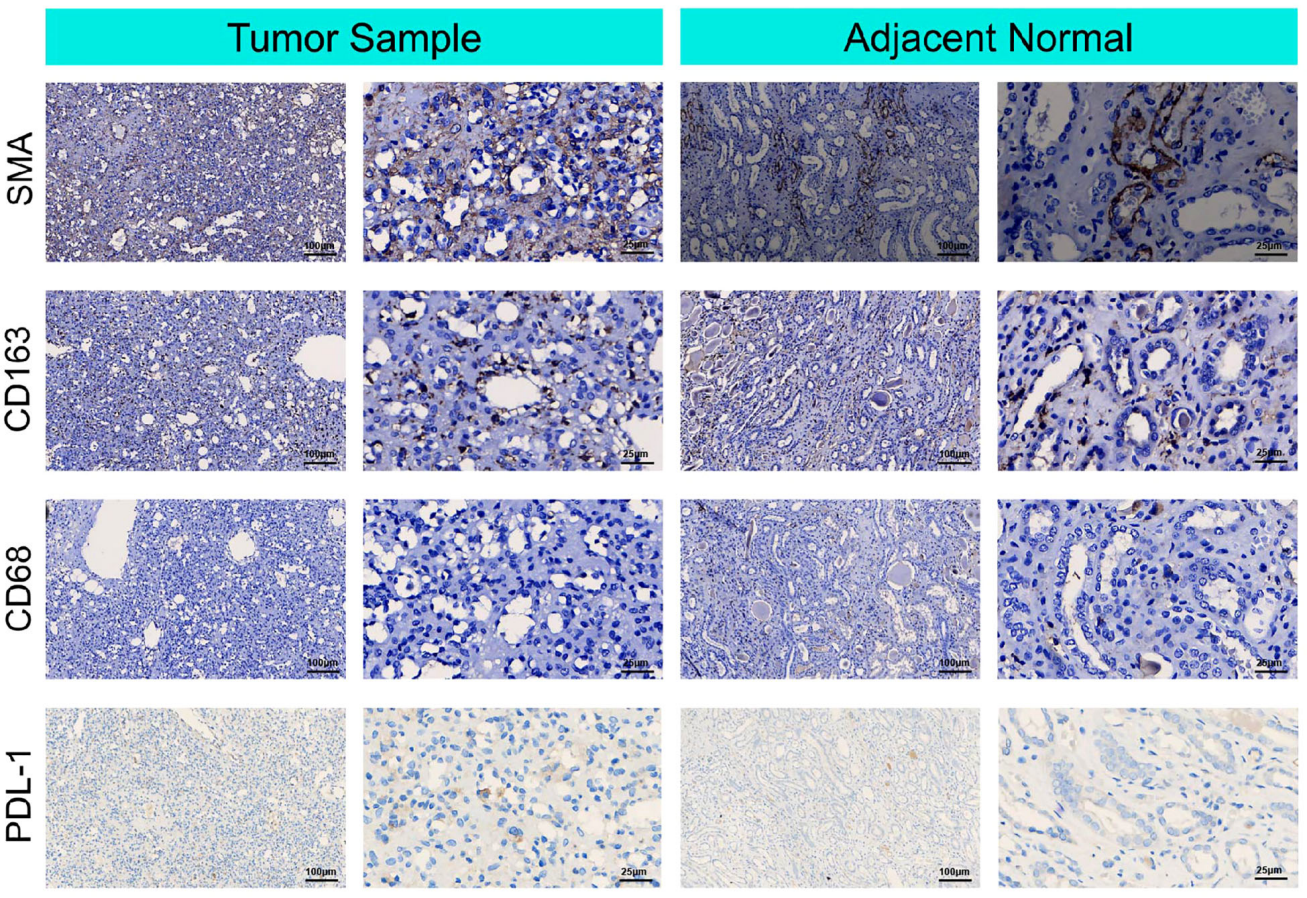
Immunohistochemical microscopic images of smooth muscle actin SMA, cluster of differentiation (CD) 168, cluster of differentiation (CD) 68 and PDL-1 for the tumor micro-environment analysis in the tumor sample and adjacent normal tissue.

For immunohistochemistry to detect components of the tumor microenvironment, we analyzed the distributions of PD-L1, markers of tumor-associated fibroblasts (SMA), markers of TAMs (CD163 and CD68), in the tumor and adjacent normal tissue. As shown in **Figure 3**, higher expression of PD-L1, CD163, and SMA was detected in the tumor tissue than in the adjacent nontumor tissue, indicating that TAMs and fibroblasts may participate in the pathogenesis of RAH.

### 3.2 Whole-genome sequencing study

#### 3.2.1 Identification of SNPs and INDELs

**Figure 4:**
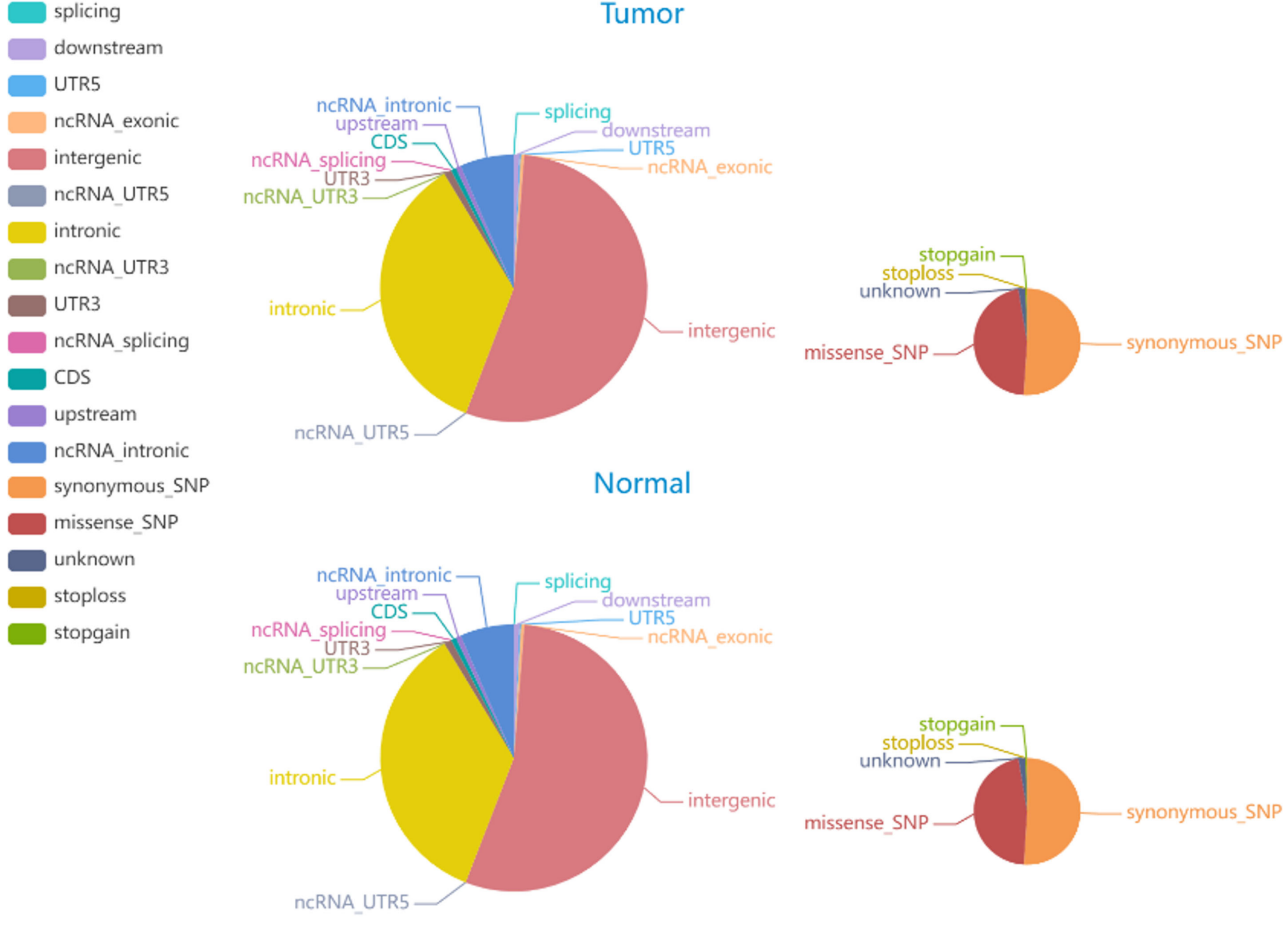
The distribution of SNPs in tumor and normal specimens is depicted in the pie chart. The left and right images display the number of SNPs in various genomic regions and the coding regions in tumor and normal specimen.

**Figure 5:**
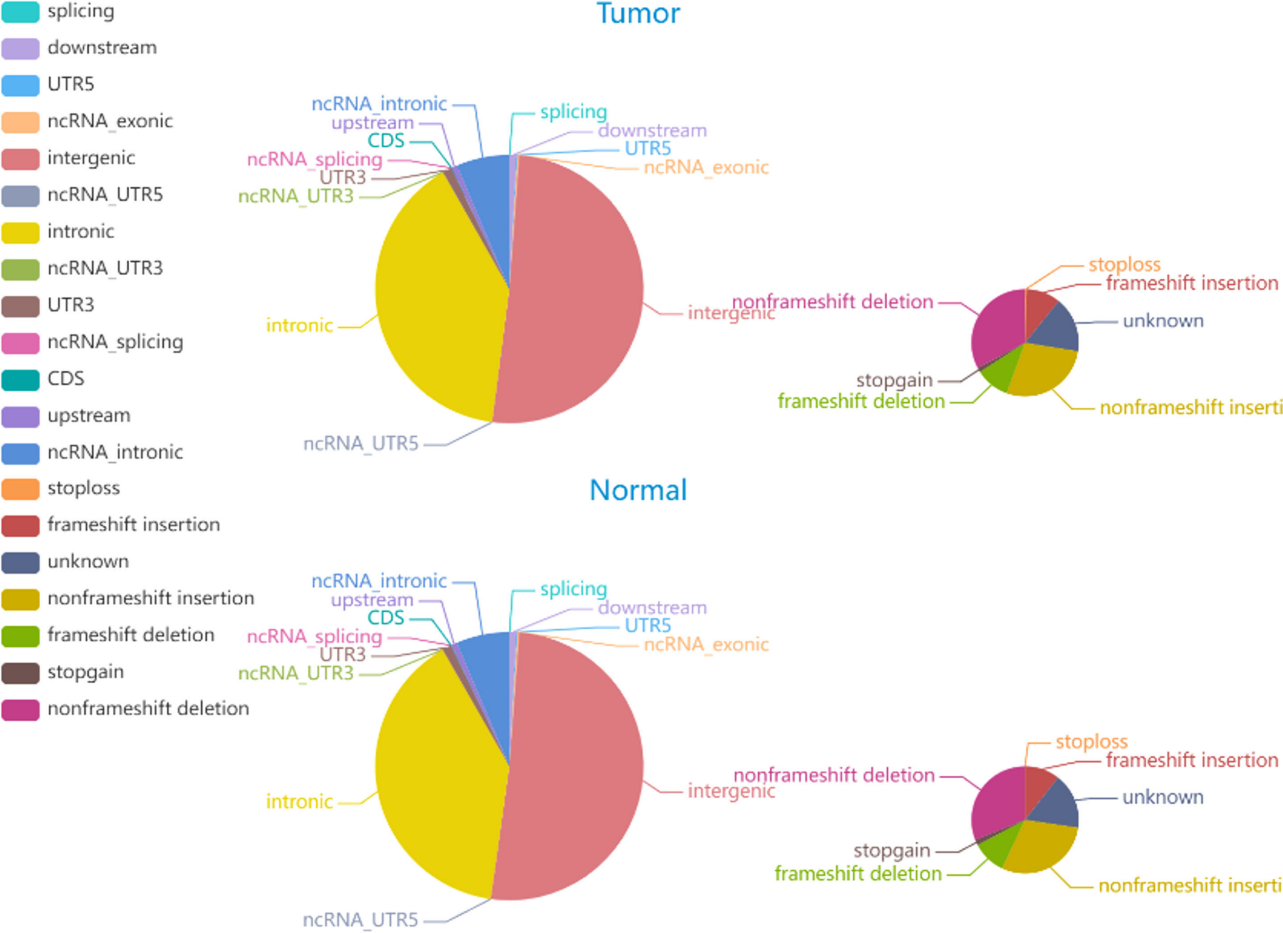
The distribution of INDELs in tumor and normal specimens is depicted in the pie chart. The left and right images display the number of INDELs in various genomic regions and the coding regions in tumor and normal specimen.

The average Q30 of the sequenced samples was 92.74%, and the average error rate was 0.03%, suggesting that the sequencing data were high quality and complied with the analytical specifications. The normal sample had a mean of 179,075,896 raw reads, and the tumor sample had a mean of 123,689,224 raw reads. The tumor sample and matched normal sample included 3,636,612 and 3,678,071 SNPs, respectively. The correctness of the SNP dataset is reflected in the transformation/inversion ratio, which is 2.05 for the tumor sample and 2.04 for the normal sample. In total, 1,053,180 and 1,061,622 INDELs were confirmed in the tumor specimen and matched normal sample, respectively. The SNP and INDEL information were displayed in **Figures 4**, **5** and **Supplementary Tables S2–S5**.

#### 3.2.2 Identification of Somatic SNVs and INDELs

Somatic mutations play a major role in the pathogenesis and evolution of tumors. A total of 6705 somatic INDELs and 10532 somatic SNVs were found, which were mainly grouped in intronic and intergenic regions. **Supplementary Tables S6, S7** contain comprehensive data on somatic SNVs and INDELs.

#### 3.2.3 Identification of SVs and CNVs

**Figure 6:**
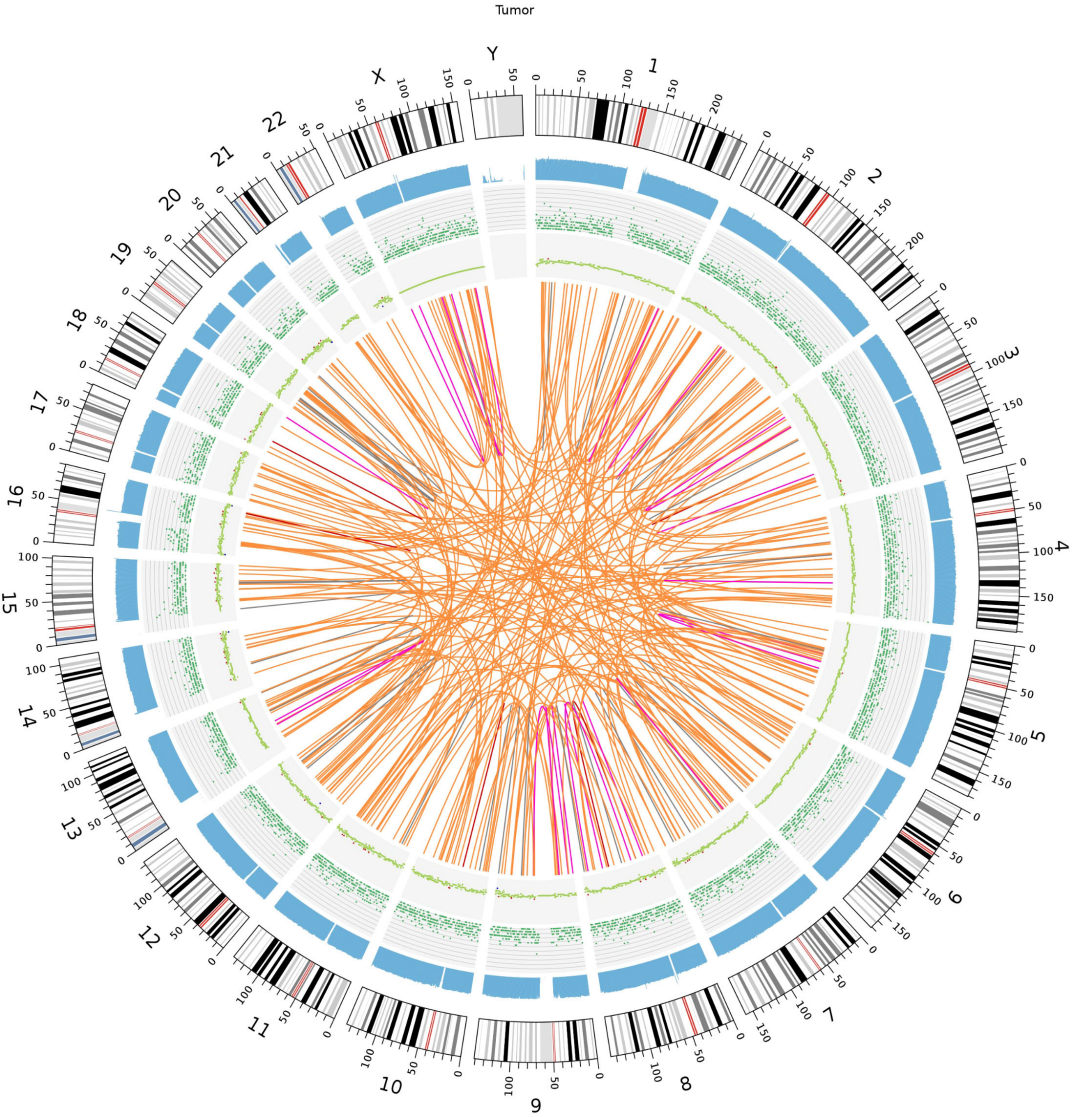
Somatic genomic variation Circo. The chromosome number, sequencing coverage map, SNPs and INDELs density, CNVs and SVs results are represented by a five-layered structure that extends from the outside to the inside.

We identified 5 duplications, 35 deletions, 187 interchromosomal translocations, and 20 intrachromosomal translocations among the somatic structural variants (SVs) of the tumor sample. There were 68 somatic copy number variants (CNVs), comprising 60 gain and 8 loss counts. **Figure 6** shows the Circos plot of somatic variation.

#### 3.2.4 Identification of Predisposing and Driver Mutating Genes

**Figure 7:**
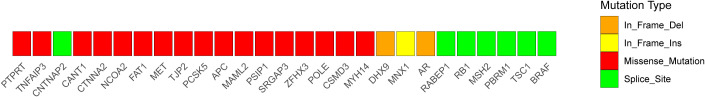
The mutational heatmaps of predisposing genes.

**Figure 8:**
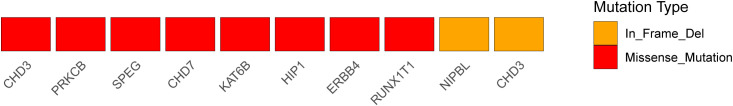
The mutational heatmaps of driver genes.

Mutations in cancer predisposition genes (CPGs) dramatically increase individuals’ risk of disease development. We identified 32 predisposing genes, such as *CNTNAP2*, *NCOA2*, *FAT1*, *MET*, *TJP2*, *PCSK5*, *MAML2*, *SRGAP3*, and *CSMD3*, the details of which are provided in **Supplementary Table S8**, the mutational heatmaps of predisposing genes was presented in **Figure 7**. Understanding the pathophysiology of cancer requires the identification of driver mutations and the genes responsible for these abnormalities, which provide tumors with a selective growth advantage. Ten driver genes mutation sites were identified, as indicated in **Supplementary Table S9**: *CHD3*, *PRKCB*, *SPEG*, *CHD7*, *KAT6B*, *HIP1*, *ERBB4*, *RUNX1T1*, *NIPBL*, and *CHD3*, presenting the mutational heatmaps of driver genes in **Figure 8**.

#### 3.2.5 Analysis of tumor purity, ploidy, and targeted drug prediction

The tumor purity was 0.18, the tumor ploidy was 1.03, and the cancer DNA fraction was 10%. On the basis of the NovoDR drug database, we identified sixteen mutation sites associated with sixty-three targeted drugs, and the detailed information is presented in **Supplementary Table S10**. There were twenty-four drugs (levonorgestrel, spironolactone, flutamide, oxandrolone, testosterone, nilutamide, fludrocortisone, drostanolone, nandrolone phenpropionate, bicalutamide, fluoxymesterone, drospirenone, danazol, testosterone propionate, boldenone, calusterone, flufenamic acid, dihydrotestosterone, methyltrienolone, cyproterone, methyltestosterone, nandrolone decanoate, androgen receptor modulators and antagonists and pi3k inhibitors) for *AR*; one drug (phosphatidylserine) for *ATP8A1*; one drug (phosphatidylserine) for *CASP1*; one drug (purvalanol a) for *CSNK1G3*; one drug (imatinib) for *DDR1*; four drugs (flt3 inhibitors, mek inhibitors, jak2 inhibitors, dot1l inhibitors) for *DEK*; fifteen drugs (lorazepam, temazepam, clobazam, alprazolam, chlordiazepoxide, clorazepate, midazolam, flurazepam, diazepam, oxazepam, triazolam, clonazepam, bromazepam, nitrazepam and venlafaxine) for *GABRQ*, one drug (niacin) for *HCAR2*, one drug (pyridoxal phosphate) for *IGSF10*, one drug (marimastat) for *MMP14*, two drugs (adenine and formycin) for MTAP, one drug (NADH) for *NADUFS6*, one drug (platinum compounds) for *PPP1R13L*, two drugs (vitamin E and dexmedetomidine) for *PRKCB*, three drugs (staurosporine, phosphonothreonine and phosphonoserine) for *PRKCQ*, and four drugs (flt3 inhibitors, MEK inhibitors, jak2 inhibitors and dot1l inhibitors) for *RUNX1T1*.

#### 3.2.6 Sanger sequencing

**Figure 9:**
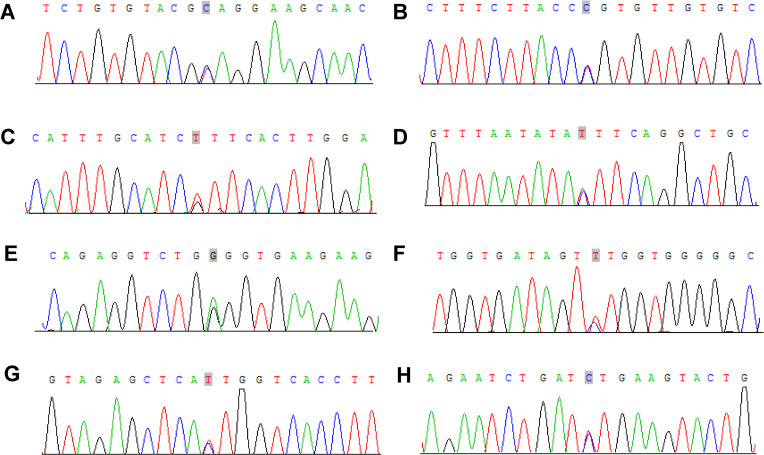
Sanger sequencing mutations electropherograms of the *CNTNAP2* at chr7: 146818068 C>T **(A)**, *NCOA2* at chr8: 71033538 C>T **(B)**, *FAT1* at chr4: 187539340 G>T **(C)**, *MET* at chr7: 116340087 C>T **(D)**, *TJP2* at chr9: 71851954 G>A **(E)**, *MAML2* at chr11: 96074675 C>T **(F)**, *SRGAP3* at chr3: 9121722 C>T **(G)**, *CSMD3* at chr8: 113347624 C>T **(H)**.

**Figure 10:**
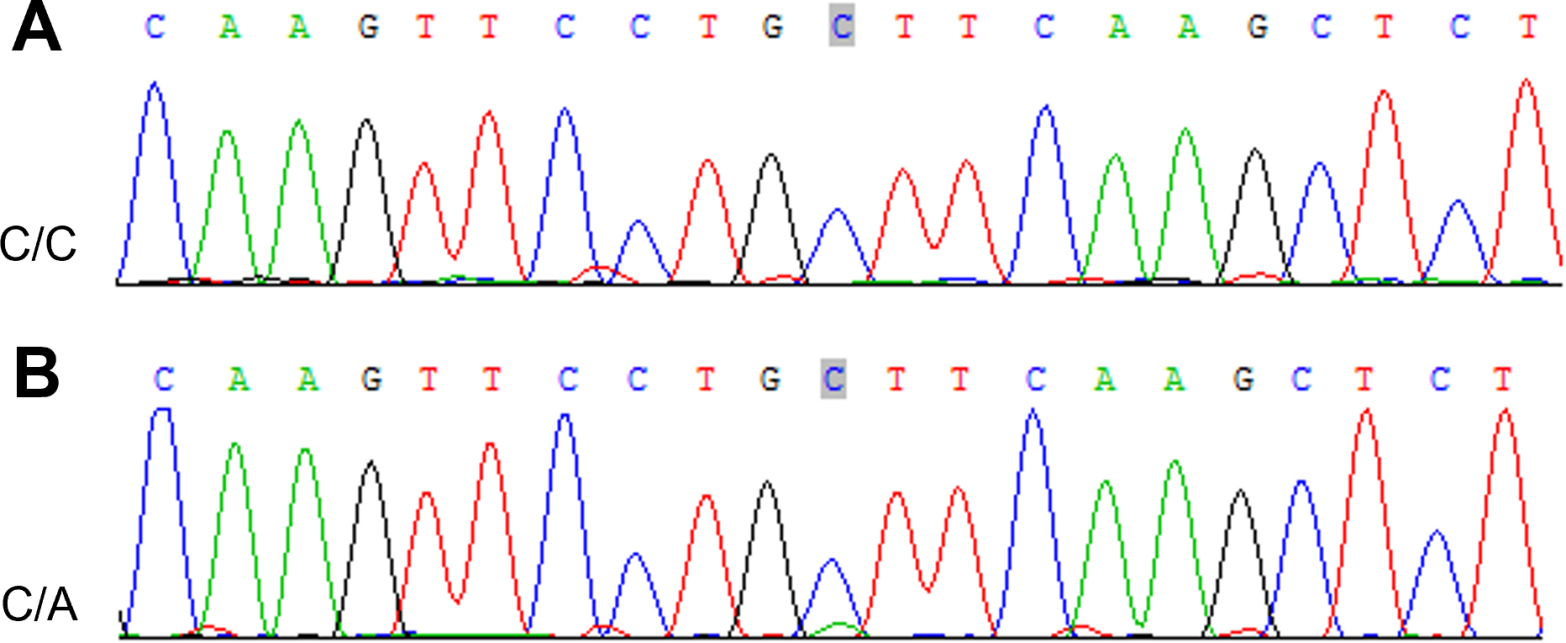
Sanger sequencing mutations electropherograms of the *HIP1* at chr7: 75186080 C>T **(A)** peripheral blood as control; **(B)** tumor.

To verify the mutations in the predisposing and driver genes, we carried out Sanger sequencing. As a result, we confirmed the presence of mutations in predisposing genes: *CNTNAP2* at chr7: 146818068 C>T, *NCOA2* at chr8: 71033538 C>T, *FAT1* at chr4: 187539340 G>T, *MET* at chr7: 116340087 C>T, *TJP2* at chr9: 71851954 G>A, *MAML2* at chr11: 96074675 C>T, *SRGAP3* at chr3: 9121722 C>T, and *CSMD3* at chr8: 113347624 C>T (**Figure 9**), while the mutation in the driver gene HIP1 at chr7: 75186080 C>T was also confirmed by Sanger sequencing (**Figure 10**).

#### 3.2.7 Protein–protein interaction network structure and functional enrichment

**Figure 11:**
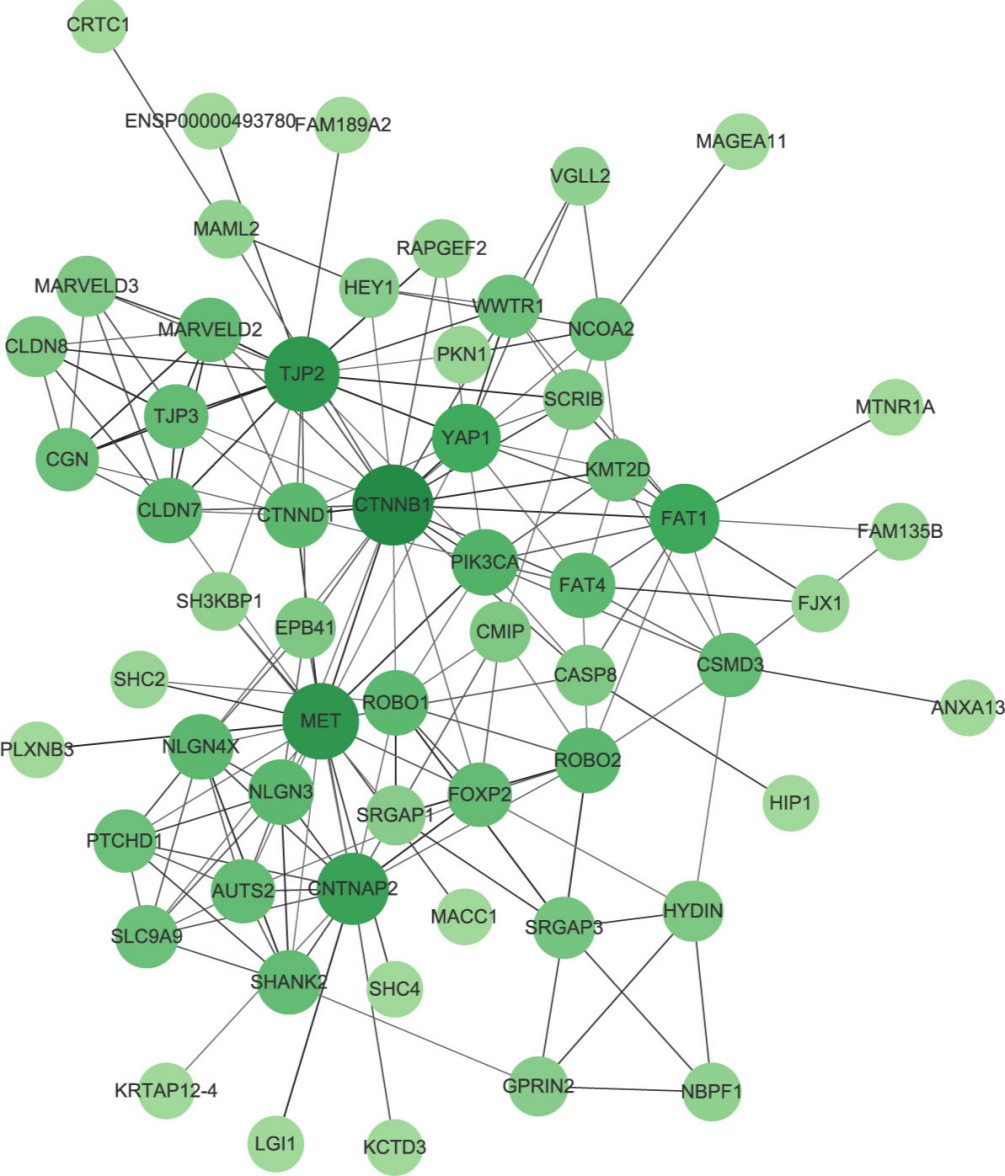
Protein–protein interaction network (PPI). The size and color of the nodes represents the degree of the nodes.

**Figure 12:**
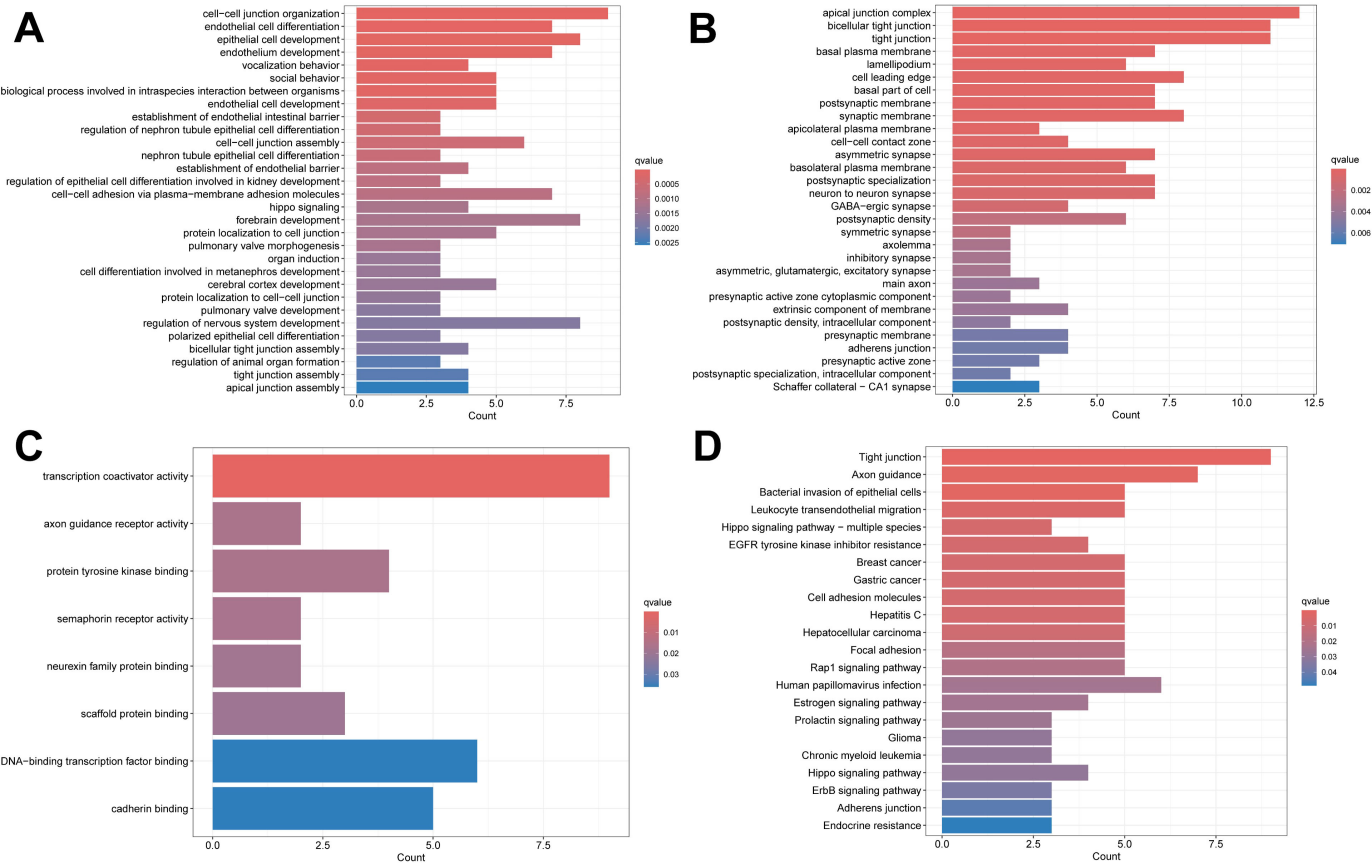
Enrichment analysis results. **(A)** The BP category of GO analysis. **(B)** The CC category of GO analysis. **(C)** The MF category of GO analysis. **(D)** KEGG pathway enrichment analysis.

The PPI network we constructed contained 59 nodes and 176 edges (**Figure 11**). According to the GO analysis, the node genes in the PPI network were significantly enriched in cell−cell junction organization and cell junction assembly in the BP category, apical junction complex in the CC category, and transcription coactivator activity in the MF category, and the genes encoding proteins involved in forming tight junctions were strongly enriched in the KEGG analysis (**Figure 12**).

#### 3.2.8 Analysis of immune cell infiltration alterations in the tumor microenvironment

**Figure 13:**
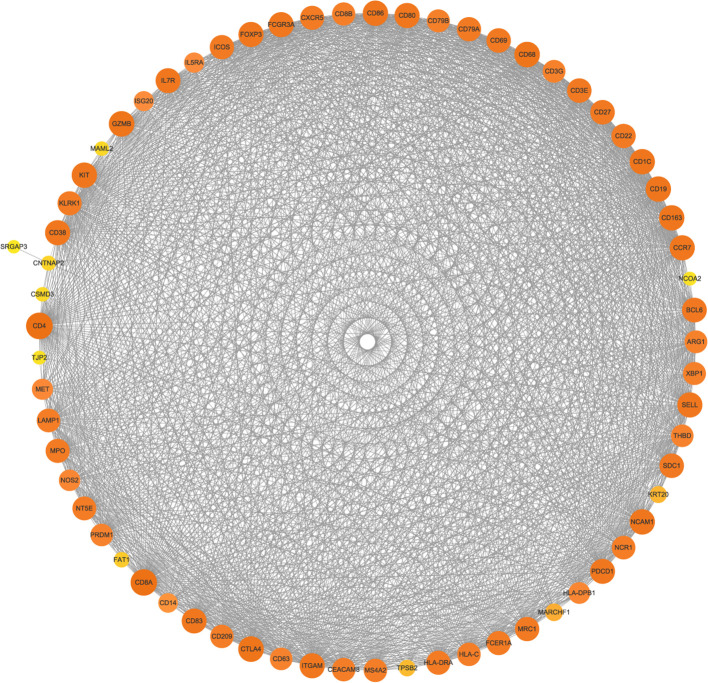
Protein–protein interaction network (PPI) of mutated genes and surface antigens of immune cells. The size and color of the nodes represents the degree of the nodes.

The PPI network of the mutated genes and surface antigens of immune cells contained 67 nodes and 1466 edges, with a PPI enrichment *p* value <1.0e-16 (**Figure 13**). The detailed results of the protein–protein interaction analysis between the mutant genes and surface antigens of immune cells (NK/T cells, mast cells, M1 macrophages, M2 macrophages and plasmacytes) are presented in **Table 1**.

**Table 1:** Protein-protein interactions analysis between mutant gene and surface antigens of immune cells in String database.

Node1	Node2	Node1 string id	Node2 string id	Co-expression	Experimentally determined interaction	Automated text mining	Combined score
CNTNAP2	NCAM1(CD56)	9606.ENSP00000354778	9606.ENSP00000480132	0.099	0.069	0.509	0.552
CSMD3	PDCD1	9606.ENSP00000297405	9606.ENSP00000335062	0	0	0.166	0.165
CSMD3	CTLA4	9606.ENSP00000297405	9606.ENSP00000497102	0	0	0.201	0.201
MET	XBP1	9606.ENSP00000317272	9606.ENSP00000216037	0	0.047	0.156	0.161
MET	NT5E	9606.ENSP00000317272	9606.ENSP00000257770	0.179	0	0.272	0.376
MET	TPSB2	9606.ENSP00000317272	9606.ENSP00000482743	0	0.181	0	0.181
MET	SELL (CD62L)	9606.ENSP00000317272	9606.ENSP00000498227	0	0.045	0.178	0.182
MET	MRC1 (CD206)	9606.ENSP00000317272	9606.ENSP00000455897	0	0.045	0.181	0.185
MET	NOS2 (iNOS)	9606.ENSP00000317272	9606.ENSP00000327251	0.104	0.045	0.169	0.227
MET	NCAM1	9606.ENSP00000317272	9606.ENSP00000480132	0	0.088	0.313	0.347
MET	SDC1 (CD138)	9606.ENSP00000317272	9606.ENSP00000370542	0.109	0	0.369	0.413
MET	PDCD1	9606.ENSP00000317272	9606.ENSP00000335062	0	0	0.432	0.432

### 3.3 Population-based study

#### 3.3.1 Clinical characteristics and pathological features

A total of 100 RAH patients were included in our study despite incomplete information on some patients, and the clinical characteristics, treatment, pathological features, and outcomes of the patients are presented in **Table 2**. With a male-to-female ratio of approximately 2:1, most patients were males, with ages ranging from 0.75 to 94 years. The presence of a renal mass was incidentally detected in most patients on examination for other diseases, while a minority of patients presented with clinical symptoms, including abdominal/flank pain (n=11), hematuria (n=7) and abdominal bulge (n=1). Among the 100 RAH patients, 46 had comorbid ESRD when RAH was detected; the cause of ESRD in 20 patients was not reported in the literature, and SLE was the most frequent cause of ESRD (n=9). Only 11 patients exhibited bilateral kidney involvement; 40 patients had unilateral kidney involvement in the left kidney, and 31 patients had unilateral kidney involvement in the right kidney. Among the 91 patients for whom treatment data were available, 71 patients underwent radical nephrectomy, 20 patients underwent partial nephrectomy, and 24 patients had cooccurring or a history of other renal tumors, including renal cell carcinoma (RCC), papillary adenomas, papillary RCC, angiomyolipoma, metanephric adenoma, acquired cystic kidney disease (ACKD), ACKD-associated RCC and Wilms’ tumor; the largest diameter of the tumors ranged from 1 mm-140 mm. Microscopic examination revealed typical capillary-sized vessels with a spleen-like sinusoidal pattern of AH, an absence of diffuse infiltrative growth and no malignant cytological features of angiosarcoma. Immunohistochemical analysis revealed that CD31, CD34 and factor VIII were positive, with a low Ki-67 labeling index. Among the patients with available follow-up data, no patients experienced disease progression or death.

**Table 2:** Clinicopathological features of renal Anastomosing Hemangiomas in the population-based study.

	Reference	Age	Gender	Clinical Manifestation	ESRD	Cause of ESRD	Diabetes	Hypertension	Site	Location	Focality	Largest Tumor Diameter (mm)	Preoperative diagnosis	Therapy	Cooccurrence/History of Renal tumor	Immunohistochemistry				Follow-up
																CD31	CD34	factor VIII	Ki-67	
1	Abboudi H 2017	62	F	Hematuria	Yes	AAV	None/NA	None/NA	Bilateral	Renal sinus/hilus/medulla	Multifocal	27	NA	Metachronous Nephrectomy	None	NA	NA	NA	NA	NA
2	Al-Maghrabi HA 2014	55	F	Left flank pain	None/NA	–	Yes	Yes	Left	Renal sinus/hilus/medulla	Unifocal	20	RCC	Partial nephrectomy	A history of right partial nephrectomy three years earlier due to clear cell RCC (Fuhrman nuclear grade II)	+	+	+	NA	NED (12 months)
3	Aravind A 2025	28	M	Abdominal pain and dyspepsia	None/NA	–	None/NA	None/NA	Left	Renal sinus/hilus/medulla and cortex	Unifocal	40	Low aggressive potential lesion	Partial nephrectomy	None	+	+	+	0.12-0.15	NED
4	Bean GR 2017 (1)	49	M	NA	None/NA	–	None/NA	None/NA	NA	NA	NA	12	NA	NA	NA	NA	NA	NA	NA	NED (9 months)
5	Bean GR 2017 (2)	53	M	Incidentally	None/NA	–	None/NA	None/NA	NA	NA	NA	33	NA	NA	NA	NA	NA	NA	NA	NED (84 months)
6	Bean GR 2017 (3)	64	M	Incidentally	None/NA	–	None/NA	None/NA	NA	NA	NA	7	NA	NA	NA	NA	NA	NA	NA	NED (107 months)
7	Berker NK 2017 (1)	24	F	Incidentally	Yes	Membranoproliferative glomerulonephritis	None/NA	None/NA	Bilateral	Renal sinus/hilus/medulla	Multifocal	30	NA	Partial nephrectomy	Papillary adenomas	+	+	+	NA	NED (10 months)
8	Berker NK 2017 (2)	57	F	Incidentally	Yes	Diabetes	Yes	None/NA	Left	Renal sinus/hilus/medulla	Unifocal	22	NA	Radical nephrectomy	NA	NA	NA	NA	NA	NED (4 months)
9	Brown JG 2010 (1)	56	M	NA	None/NA	–	None/NA	None/NA	Right	NA	NA	13	NA	Partial nephrectomy	None	NA	NA	NA	NA	NA
10	Brown JG 2010 (2)	33	F	NA	None/NA	–	None/NA	None/NA	Left	NA	NA	32	NA	Radical nephrectomy	None	NA	NA	NA	NA	NA
11	Brown JG 2010 (3)	21	M	Incidently	Yes	None/NA	None/NA	None/NA	Right	NA	NA	22	NA	NA	None	NA	NA	NA	NA	NED (24 months)
12	Brown JG 2010 (4)	44	F	NA	None/NA	–	None/NA	None/NA	Left	NA	NA	20	NA	NA	None	NA	NA	NA	NA	NED (72 months)
13	Brown JG 2010 (5)	83	F	NA	None/NA	–	None/NA	None/NA	Left	NA	NA	35	NA	NA	None	NA	NA	NA	NA	NED (24 months)
14	Buttner M 2013 (1)	33	M	NA	Yes	NA	None/NA	None/NA	NA	Renal sinus/hilus/medulla and cortex	Multifocal	7	NA	Radical nephrectomy	None	NA	NA	NA	NA	NA
15	Buttner M 2013 (2)	70	M	NA	Yes	NA	None/NA	None/NA	NA	Renal sinus/hilus/medulla	Unifocal	3	NA	Radical nephrectomy	Papillary adenoma	NA	NA	NA	NA	NA
16	Buttner M 2013 (3)	55	M	NA	Yes	NA	None/NA	None/NA	NA	Renal cortex	Unifocal	2	NA	Radical nephrectomy	None	NA	NA	NA	NA	NA
17	Buttner M 2013 (4)	42	M	NA	Yes	NA	None/NA	None/NA	NA	Renal sinus/hilus/medulla and cortex	Multifocal	1.5	NA	Radical nephrectomy	Papillary adenoma, cRCC	NA	NA	NA	NA	NA
18	Buttner M 2013 (5)	32	M	NA	Yes	NA	None/NA	None/NA	NA	Renal cortex	Multifocal	6	NA	Radical nephrectomy	Papillary RCC, angiomyolipoma	NA	NA	NA	NA	NA
19	Buttner M 2013 (6)	42	M	NA	Yes	NA	None/NA	None/NA	NA	Renal sinus/hilus/medulla	Unifocal	3	NA	Radical nephrectomy	None	NA	NA	NA	NA	NA
20	Buttner M 2013 (7)	45	F	NA	Yes	NA	None/NA	None/NA	NA	Renal sinus/hilus/medulla and cortex	Multifocal	25	NA	Radical nephrectomy	None	NA	NA	NA	NA	NA
21	Buttner M 2013 (8)	44	F	NA	Yes	NA	None/NA	None/NA	NA	Renal sinus/hilus/medulla	Unifocal	1	NA	Radical nephrectomy	Papillary RCC, metanephric adenoma	NA	NA	NA	NA	NA
22	Caballes AB 2019	10	M	A firm and painless bulge at the left paraumbilical area	None/NA	–	None/NA	None/NA	Left	Renal sinus/hilus/medulla	Unifocal	120	Wilms’ tumor	Radical nephrectomy	None	NA	+	NA	0	NA
23	Capinha MD 2023	70	M	Incidentally	None/NA	–	None/NA	Yes	Left	Renal sinus/hilus/medulla	Unifocal	20	RCC	Radical nephrectomy	None	+	NA	NA	NA	NED (18 months)
24	Cha JS 2016	43	M	Incidentally	None/NA	–	Yes	None/NA	Right	Renal sinus/hilus/medulla	Unifocal	43	RCC	Radical nephrectomy	None	+	+	+	NA	NED (5 months)
25	Chandran N 2019	36	M	Incidentally	Yes	NA	None/NA	None/NA	Bilateral	Renal sinus/hilus/medulla	Multifocal	26	RCC	Radical nephrectomy	None	NA	+	NA	NA	NA
26	Chen J 2024	59	M	Incidentally	None/NA	–	None/NA	None/NA	Left	Renal sinus/hilus/medulla	Unifocal	22	RCC	Radical nephrectomy	RCC	+	+	NA	0.02-0.05	NED
27	Cheon PM 2018	40	M	Incidentally	None/NA	–	None/NA	None/NA	Left	Renal sinus/hilus/medulla	Unifocal	53	RCC	Radical nephrectomy	None	+	+	+	0.2	NED (1 months)
28	Chou S 2014 (1)	50	F	Incidentally	Yes	–	None/NA	None/NA	Left	Renal sinus/hilus/medulla	Unifocal	10	NA	Radical nephrectomy	NA	+	+	NA	NA	NA
29	Chou S 2014 (2)	60	M	Incidentally	Yes	–	None/NA	Yes	Left	Renal sinus/hilus/medulla	Multifocal	18	NA	Radical nephrectomy	NA	+	+	NA	NA	NA
30	Chua WM 2022	32	F	Incidentally	Yes	SLE	None/NA	None/NA	Left	Renal sinus/hilus/medulla	Multifocal	NA	Neuroendocrine tumor	NA	None	+	NA	NA	NA	NA
31	Downes MR 2014 (1)	59	F	Incidentally	None/NA	–	None/NA	None/NA	Left	Renal sinus/hilus/medulla	Unifocal	45	RCC	Radical nephrectomy	NA	+	+	+	NA	NA
32	Downes MR 2014 (2)	28	M	Incidentally	None/NA	–	None/NA	None/NA	Right	Renal sinus/hilus/medulla	Unifocal	13	NA	Ultrasound guided kidney core biopsy	History of left RCC	+	+	NA	NA	NA
33	Faraz M 2025 (1)	52	M	Incidentally	None/NA	–	None/NA	None/NA	Left	NA	Unifocal	28	NA	Partial nephrectomy	None	+	NA	+	NA	DWOD (81months)
34	Faraz M 2025 (2)	71	F	Incidentally	None/NA	–	None/NA	None/NA	Left	NA	Unifocal	28	NA	Radical nephrectomy	None	+	NA	NA	NA	NED (83 months)
35	Faraz M 2025 (3)	69	M	Incidentally	None/NA	–	None/NA	None/NA	Left	NA	Unifocal		NA	Partial nephrectomy	None	NA	NA	NA	NA	NED (35 months)
36	Faraz M 2025 (4)	94	M	Incidentally	None/NA	–	None/NA	None/NA	Right	NA	Unifocal		NA	Biopsy	None	NA	NA	NA	NA	NED (31 months)
37	Faraz M 2025 (5)	47	F	Incidentally	None/NA	–	None/NA	None/NA	Left	NA	Unifocal		NA	Radical nephrectomy	None	NA	+	NA	NA	NED (161 months)
38	Faraz M 2025 (6)	76	M	Incidentally	None/NA	–	None/NA	None/NA	NA	NA	Unifocal		NA	Partial nephrectomy	None	NA	NA	NA	NA	NA
39	Faraz M 2025 (7)	45	M	Incidentally	None/NA	–	None/NA	None/NA	Left	NA	Unifocal		NA	Radical nephrectomy	None	+	+	NA	NA	NED (156 months)
40	Faraz M 2025 (8)	59	M	Incidentally	None/NA	–	None/NA	None/NA	Left	NA	Unifocal		NA	Partial nephrectomy	None	NA	NA	NA	NA	NED (108 months)
41	Faraz M 2025 (9)	56	F	Incidentally	None/NA	–	None/NA	None/NA	Left	NA	Multifocal		NA	Radical nephrectomy	AMLEC, AML, cortical cysts	NA	NA	NA	NA	NED (24 months)
42	Faraz M 2025 (10)	67	F	Incidentally	None/NA	–	None/NA	None/NA	Right	NA	Unifocal		NA	Partial nephrectomy	ccRCC	+	NA	NA	NA	NED (9 months)
43	Faraz M 2025 (11)	41	M	Incidentally	None/NA	–	None/NA	None/NA	Right	NA	Unifocal		NA	Partial nephrectomy	None	+	NA	NA	NA	NED (81 months)
44	Faraz M 2025 (12)	62	M	Incidentally	Yes	None/NA	None/NA	None/NA	Left	NA	Multifocal		NA	Radical nephrectomy	None	+	NA	NA	NA	NED (46 months)
45	Faraz M 2025 (13)	50	F	Incidentally	Yes	None/NA	None/NA	None/NA	Left	NA	Multifocal		NA	Radical nephrectomy	None	+	NA	NA	NA	NA
46	Faraz M 2025 (14)	80	M	Incidentally	None/NA	–	None/NA	None/NA	Right	NA	Unifocal		NA	Partial nephrectomy	None	+	NA	NA	NA	NED (24 months)
47	Faraz M 2025 (15)	70	M	Incidentally	None/NA	–	None/NA	None/NA	Right	NA	Unifocal		NA	Partial nephrectomy	None	+	+	NA	NA	NED (4 months)
48	Faraz M 2025 (16)	33	F	Left flank painand gross hematuria	None/NA	–	None/NA	None/NA	Right	NA	Unifocal		NA	Radical nephrectomy	None	+	+	NA	NA	NA
49	Faraz M 2025 (17)	34	M	Incidentally	Yes	–	None/NA	None/NA	Bilateral	NA	Multifocal		NA	Partial nephrectomy	AML, transplant kidneys	NA	NA	NA	NA	NED (5 months)
50	Faraz M 2025 (18)	50	M	Incidentally	None/NA	–	None/NA	None/NA	Left	NA	Unifocal		NA	Partial nephrectomy	None	+	NA	NA	NA	NED (72 months)
51	Gong C 2024	58	F	Pain in the area of right kidney	None/NA	–	None/NA	None/NA	Left	Renal sinus/hilus/medulla	Unifocal	25	NA	Radical nephrectomy	None	NA	NA	NA	NA	NA
52	Heidegger I 2014	56	M	Incidentally	None/NA	–	None/NA	None/NA	Right	Renal sinus/hilus/medulla	Unifocal	50	RCC	Radical nephrectomy	None	+	+	NA	<1%	NED (128 month)
53	Johnstone KJ 2020	70	M	Incidentally	None/NA	–	None/NA	None/NA	Right	Renal sinus/hilus/medulla and cortex	Multifocal	35	NA	Radical nephrectomy	None	+	+	+	Low	NA
54	Kim CS 2021	35	F	Incidentally	Yes	SLE	None/NA	Yes	Right	Renal sinus/hilus/medulla	Unifocal	17	RCC	Radical nephrectomy	None	+	+	NA	NA	NA
55	Kryvenko ON 2011 (1)	51	F	Incidentally	Yes	None/NA	None/NA	None/NA	Right	Renal sinus/hilus/medulla	Unifocal	10	NA	Radical nephrectomy	NA	NA	+	NA	NA	NED (7 months)
56	Kryvenko ON 2011 (2)	39	M	Incidentally	None/NA	None/NA	None/NA	Yes	Right	Renal sinus/hilus/medulla and cortex	Unifocal	50	NA	Radical nephrectomy	NA	NA	+	NA	NA	NED (122 months)
57	Kryvenko ON 2011 (3)	54	F	Incidentally	Yes	None/NA	None/NA	None/NA	Bilateral	Renal sinus/hilus/medulla and cortex	Multifocal	12	NA	Radical nephrectomy	NA	NA	+	NA	NA	NED (3 months)
58	Kryvenko ON 2014 (1)	68	F	Gross haematuria	Yes	Diabetes	Yes	None/NA	Right	NA	Multifocal	15	NA	Radical nephrectomy	None	NA	NA	NA	NA	NA
59	Kryvenko ON 2014 (2)	51	F	Incidentally	Yes	NA	None/NA	None/NA	Right	NA	Unifocal	10	NA	Radical nephrectomy	ACKD	NA	NA	NA	NA	NA
60	Kryvenko ON 2014 (3)	54	F	Incidentally	Yes	Hypertension	None/NA	Yes	Bilateral	NA	Multifocal	11	NA	Radical nephrectomy	ccRCC, papillary adenoma	NA	NA	NA	NA	NA
61	Kryvenko ON 2014 (4)	29	M	Incidentally	Yes	Focal segmental glomerulosclerosis	None/NA	None/NA	Bilateral	NA	Multifocal	13	NA	Radical nephrectomy	None	NA	NA	NA	NA	NA
62	Kryvenko ON 2014 (5)	40	M	Incidentally	Yes	SLE	None/NA	None/NA	Left	NA	Unifocal	2.5	NA	Radical nephrectomy	ACKD, Clear cell papillary RCC, ACKD- associated RCC, papillary adenoma	NA	NA	NA	NA	NA
63	Kryvenko ON 2014 (6)	34	M	Abdominal pain, haematuria, retroperitoneal haematoma	Yes	SLE	None/NA	None/NA	Right	NA	Multifocal	13	NA	Radical nephrectomy	ACKD, ACKD- associated RCC	NA	NA	NA	NA	NA
64	Kryvenko ON 2014 (7)	62	M	Incidentally	Yes	Hypertension	None/NA	Yes	Left	NA	Unifocal	7	NA	Radical nephrectomy	ACKD, ACKD-associated RCC, ACKD-associated RCC precursor	NA	NA	NA	NA	NA
65	Kryvenko ON 2014 (8)	40	M	Incidentally	Yes	SLE	None/NA	None/NA	Left	NA	Multifocal	28	NA	Radical nephrectomy	None	NA	NA	NA	NA	NA
66	Kryvenko ON 2014 (9)	46	M	Incidentally	Yes	IgA nephropathy	None/NA	None/NA	Left	NA	Unifocal	16	NA	Radical nephrectomy	None	NA	NA	NA	NA	NA
67	Kryvenko ON 2014 (10)	60	M	Incidentally	Yes	Hereditary nephritis	None/NA	None/NA	Left	NA	Unifocal	12	NA	Radical nephrectomy	ACKD, ACKD-associated RCC precursor, papillary adenoma	NA	NA	NA	NA	NA
68	Kryvenko ON 2014 (11)	49	M	Microscopic haematuria	Yes	Focal segmental glomerulosclerosis	None/NA	None/NA	Right	NA	Unifocal	35	NA	Radical nephrectomy	None	NA	NA	NA	NA	NA
69	Kryvenko ON 2014 (12)	49	M	NA	Yes	Hypertension	None/NA	Yes	Left	NA	Unifocal	13	NA	Radical nephrectomy	ACKD	NA	NA	NA	NA	NA
70	Kryvenko ON 2014 (13)	66	M	Flank pain	Yes	Hypertension	None/NA	Yes	Right	NA	Unifocal	30	NA	Radical nephrectomy	ACKD, ACKD-associated RCC precursor, papillary adenoma	NA	NA	NA	NA	NA
71	Kryvenko ON 2014 (14)	15	M	Incidentally	Yes	Focal segmental glomerulosclerosis	None/NA	None/NA	Bilateral	NA	Multifocal	7	NA	Radical nephrectomy	ACKD	NA	NA	NA	NA	NA
72	Kryvenko ON 2014 (15)	0.75	M	Incidentally	Yes	Congenital nephroticsyndrome	None/NA	None/NA	Right	NA	Unifocal	10	NA	Radical nephrectomy	Wilms’ tumour	NA	NA	NA	NA	NA
73	Kryvenko ON 2014 (16)	17	M	Abdominal pain, haematuria, retroperitoneal haematoma	Yes	FSGS secondary tominimal changedisease	None/NA	None/NA	Bilateral	NA	Multifocal	28	NA	Radical nephrectomy	ACKD, Unclassified RCC, Papillary adenomas	NA	NA	NA	NA	NA
74	Lo CH 2021	84	M	Incidentally	None/NA	None/NA	None/NA	Yes	Left	Renal sinus/hilus/medulla	Unifocal	55	Cystic RCC	Radical nephrectomy	None	+	NA	NA	<10%	Dead of aspiration pneumonia (2 weeks)
75	Lobo J 2017	63	M	Hematuria and dysuria	None/NA	None/NA	None/NA	None/NA	Right	Renal sinus/hilus/medulla	Unifocal	50	Urothelial neoplasm	Radical nephrectomy	None	NA	NA	NA	<1%	NA
76	Manohar V 2020	40	F	Vague upper abdominal pain	None/NA	None/NA	None/NA	None/NA	Left	Renal sinus/hilus/medulla	Unifocal	140	RCC	Radical nephrectomy	None	+	+	NA	NA	NED (24 months)
77	Mehta V 2012 (1)	49	M	NA	Yes	None/NA	None/NA	None/NA	NA	NA	NA	20	NA	Radical nephrectomy	None	NA	NA	NA	NA	NED (3 months)
78	Mehta V 2012 (2)	55	M	NA	Yes	None/NA	None/NA	None/NA	NA	NA	NA	6	NA	Radical nephrectomy	Papillary adenomas	NA	NA	NA	NA	NED (3 months)
79	Mehta V 2012 (3)	45	M	NA	Yes	None/NA	None/NA	None/NA	NA	NA	NA	19	NA	Radical nephrectomy	None	NA	NA	NA	NA	NED (12 months)
80	Montgomery E 2009 (1)	74	M	NA	NA	None/NA	None/NA	None/NA	NA	NA	NA	15	NA	Radical nephrectomy	NA	NA	NA	NA	NA	NED (36 months)
81	Montgomery E 2009 (2)	75	F	NA	NA	None/NA	None/NA	None/NA	NA	NA	NA	20	NA	Radical nephrectomy	NA	NA	NA	NA	NA	NA
82	Montgomery E 2009 (3)	49	M	NA	NA	None/NA	None/NA	None/NA	NA	Renal sinus/hilus/medulla	NA	13	NA	Radical nephrectomy	NA	NA	NA	NA	NA	NED (12 months)
83	Omiyale AO 2015	64	M	Incidentally	None/NA	None/NA	Yes	None/NA	Left	Renal sinus/hilus/medulla	Unifocal	24	RCC	Radical nephrectomy	None	+	+	NA	NA	NED (10 months)
84	Pantelides NM 2012	57	F	Incidentally	Yes	AAV	None/NA	None/NA	Right	Renal sinus/hilus/medulla	Unifocal	27	NA	Radical nephrectomy	None	NA	NA	NA	NA	NA
85	Patel SR 2019	39	M	Incidentally	Yes	SLE	None/NA	None/NA	Bilateral	Renal sinus/hilus/medulla	Multifocal	15	AH	Radical nephrectomy	None	NA	NA	NA	NA	NA
86	Perdiki M 2017 (1)	64	F	Back pain	None/NA	None/NA	None/NA	None/NA	Right	Renal sinus/hilus/medulla and cortex	Unifocal	11	NA	Partial nephrectomy	NA	+	+	NA	NA	NED (25 months)
87	Perdiki M 2017 (2)	47	M	Incidentally	Yes	SLE	None/NA	None/NA	Left	Renal sinus/hilus/medulla and cortex	Multifocal	28	NA	Radical nephrectomy	NA	+	+	NA	NA	NED (14 months)
88	Rubio Fernández A 2015	41	M	Incidentally	Yes	SLE	None/NA	None/NA	Bilateral	Renal sinus/hilus/medulla	Multifocal	80	Malignant renal tumor	Radical nephrectomy	None	+	+	NA	NA	NA
89	Rupanshu 2023	35	F	Incidentally	Yes	SLE	None/NA	None/NA	Right	NA	Unifocal	17	RCC	Radical nephrectomy	None	+	+	NA	NA	NED (NA)
90	Sasaki Y 2022	65	M	Incidentally	None/NA	None/NA	None/NA	None/NA	Right	Renal cortex	Unifocal	16	AH	Partial nephrectomy	None	+	+	+	0	NED (3 months)
91	Silva MA 2017	53	M	Incidentally	None/NA	None/NA	None/NA	None/NA	Left	Renal cortex	Unifocal	NA	Complex renal cystic lesion	Partial nephrectomy	None	NA	NA	NA	NA	NA
92	Tahir M 2016	57	M	Incidentally	None/NA	None/NA	None/NA	None/NA	Left	Renal sinus/hilus/medulla	Unifocal	30	NA	Radical nephrectomy	None	+	+	+	NA	NA
93	Tao LL 2014	32	M	Incidentally	None/NA	None/NA	None/NA	None/NA	Left	Renal sinus/hilus/medulla	Unifocal	26	NA	Radical nephrectomy	None	+	+	NA	0	NA
94	Tran TA 2012	61	M	Right-sided back pain with radiation to his right hip	None/NA	None/NA	None/NA	None/NA	Right	Renal sinus/hilus/medulla and cortex	Unifocal	24	NA	Radical nephrectomy	None	+	NA	+	NA	NA
95	Veerwal A 2020	25	M	Right fiank pain	None/NA	None/NA	None/NA	None/NA	Right	NA	Unifocal	NA	RCC	Radical nephrectomy	None	NA	NA	NA	NA	NA
96	Wetherell DR 2013	74	M	Incidentally	None/NA	None/NA	None/NA	None/NA	Right	Renal sinus/hilus/medulla	Unifocal	50	RCC	Radical nephrectomy	None	+	+	+	NA	Dead (6 weeks)
97	Zhang W 2015	25	F	Incidentally	None/NA	None/NA	None/NA	None/NA	Right	Renal sinus/hilus/medulla	Unifocal	12	NA	Partial nephrectomy	None	+	+	NA	NA	NED (16 months)
98	Zhang X 2023	61	M	Incidentally	None/NA	None/NA	None/NA	None/NA	Left	Renal sinus/hilus/medulla and cortex	Unifocal	18	NA	Radical nephrectomy	None	+	+	NA	0.1	NED (13 months)
99	Zhao M 2013	48	M	Incidentally	None/NA	None/NA	None/NA	None/NA	Right	Renal cortex	Unifocal	25	RCC	Partial nephrectomy	None	+	+	NA	NA	NED (12 months)
100	Our study	71	F	Incidentally	None/NA	None/NA	Yes	Yes	Left	Renal sinus/hilus/medulla	Unifocal	24	NA	Partial nephrectomy	NA	+	+	NA	0.1	NED (16 months)

ESRD, end stage renal disease; M, male; F, female; RCC, renal cell carcinoma; NED, no evidence of disease; DWOD, died without disease; AH, anastomosing hemangioma; ACKD, acquired cystic kidney disease; AAV, ANCA-associated vasculitis; SLE, systemic lupus erythematosus; NA, not available.

#### 3.3.2 Comparison of RAHs with ESRD and RAHs without ESRD

**Figure 14:**
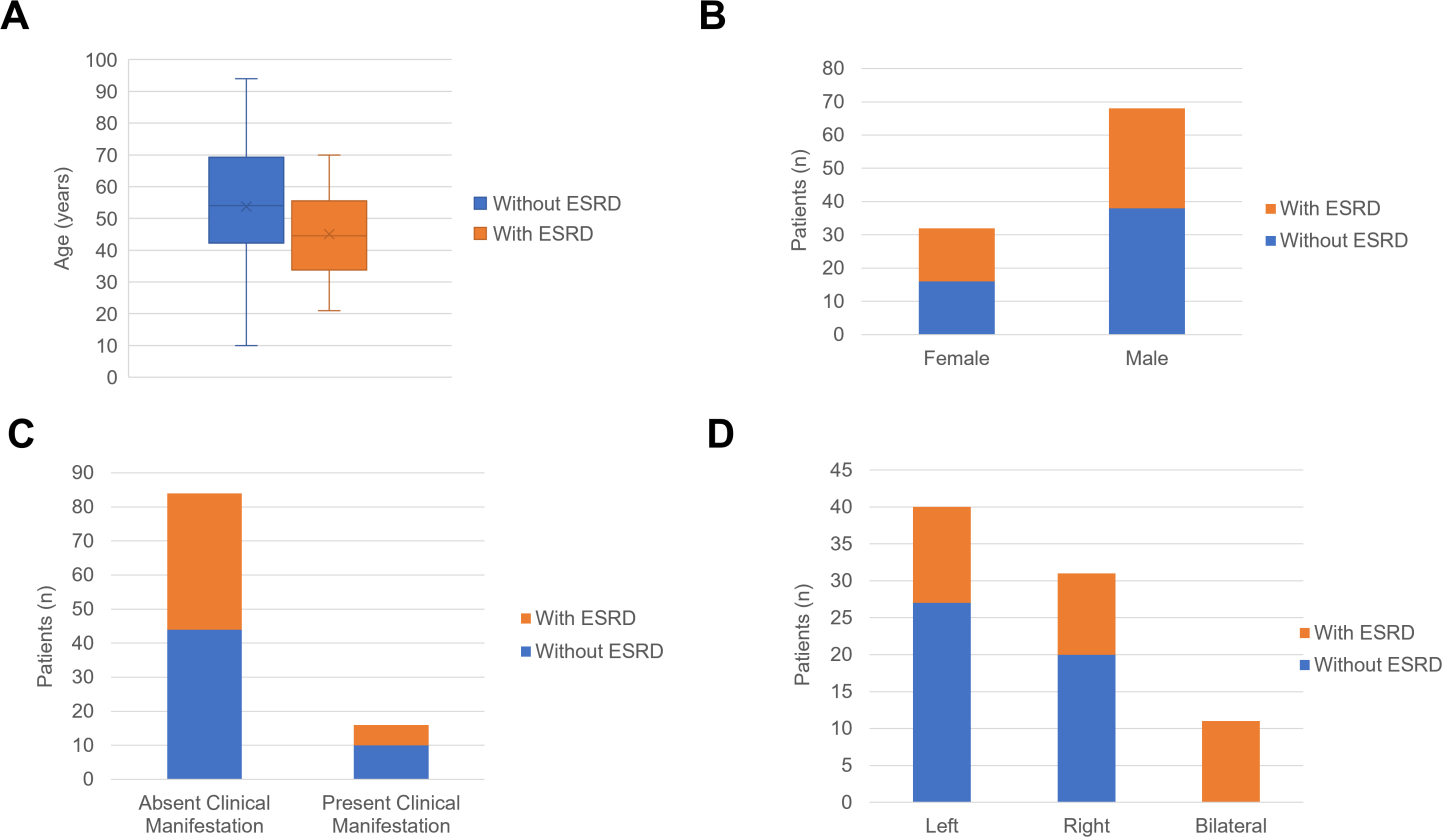
**(A)** The boxplots of age in the patients with end-stage renal disease (ESRD) and patients without ESRD. **(B)** The column charts of patients with ESRD and patients without ESRD in the female and male patients. **(C)** The column charts of patients with ESRD and patients without ESRD in the patients absent clinical manifestation and patients present clinical manifestation. **(D)** The column charts of patients with ESRD and patients without ESRD in the female with tumor in the left kidney, right kidney and bilateral kidney.

**Figure 15:**
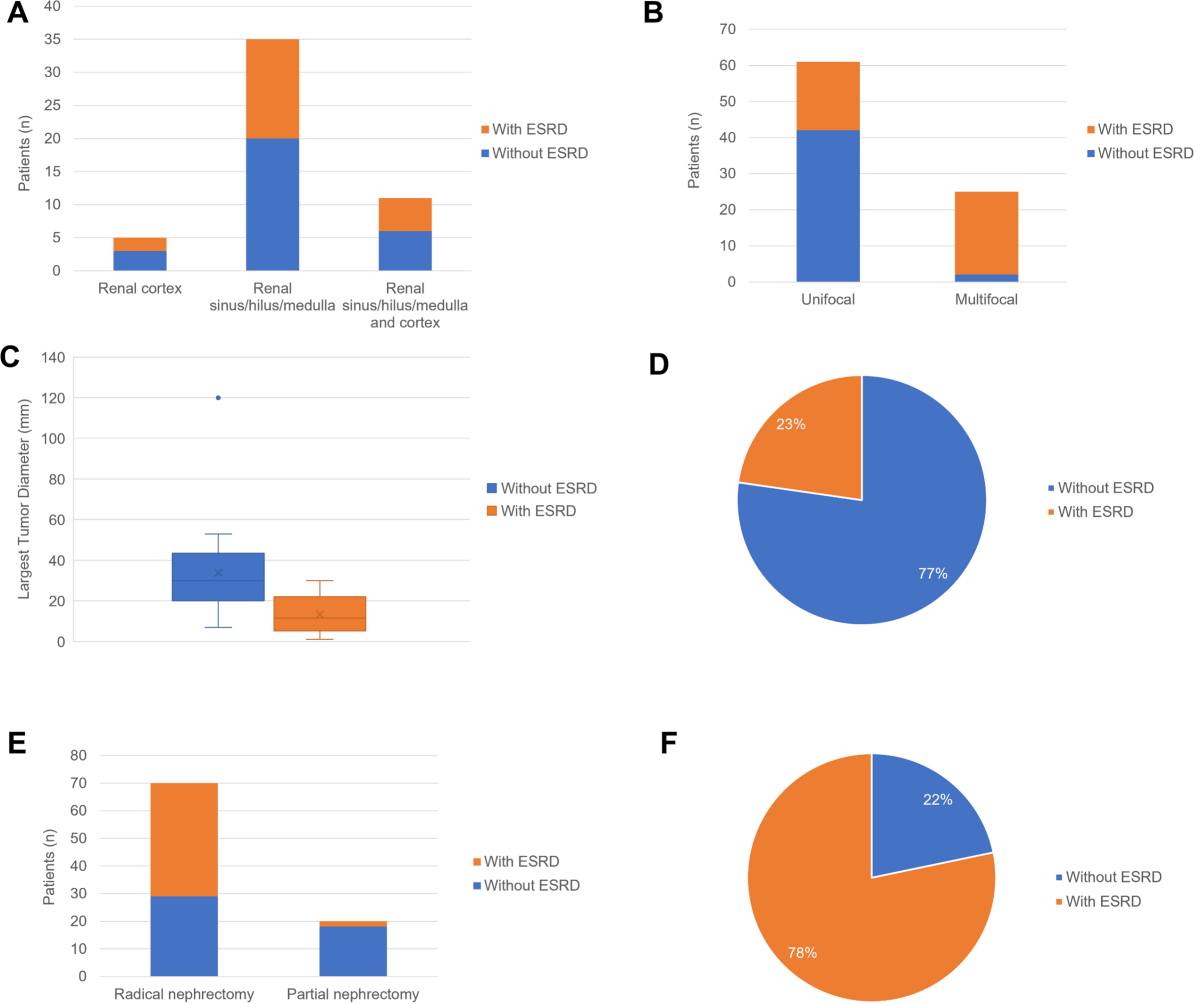
**(A)** The column charts of patients with ESRD and patients without ESRD in the female with tumor arising from renal cortex, renal sinus/hilus/medulla and renal sinus/hilus/medulla and cortex. **(B)** The column charts of patients with ESRD and patients without ESRD in patients with unifocal tumor and multifocal tumors. **(C)** The boxplots of largest tumor diameter in the patients with end-stage renal disease (ESRD) and patients without ESRD. **(D)** The pie chart of patients with ESRD and patients without ESRD in the patients with preoperative misdiagnosis. **(E)** The column charts of patients with ESRD and patients without ESRD in patients underwent partial nephrectomy and radical nephrectomy. **(F)** The pie chart of patients with ESRD and patients without ESRD in the patients with cooccurrence/History of other renal tumors.

In the comparison of RAHs with ESRD and RAHs without ESRD, as shown in **Table 3** and **Figures 14**, **15**, age, tumor site, tumor focality, largest tumor diameter and surgical approach were significantly different (*p*<0.05). Patients with ESRD and RAHs without RSRD were diagnosed at a younger age than those with RAHs without RSRD. Moreover, smaller bilateral and multifocal tumors were more prevalent among RAHs with ESRD.

**Table 3:** Comparison of features of renal Anastomosing Hemangiomas between patients with end-stage renal disease (ESRD) and patients without ESRD.

	All patients (n=100)	With ESRD (n=46)	Without ESRD (n=54)	*p*
Age (Mean ± SD)	50.32 ± 16.96	44.34 ± 14.73	55.41 ± 17.19	**0.001**
Gender (n, %)				
Female	32 (32.0%)	16 (34.8%)	16 (29.6%)	0.582
Male	68 (68.0%)	30 (65.2%)	38 (70.5%)	
Clinical Manifestation (n, %)				
Absent	83 (83.0%)	40 (87.0%)	43 (79.6%)	0.363
Present	17 (17.0%)	6 (13.0%)	11 (20.4%)	
Site (n, %)				
Left	40 (40.0%)	13 (28.3%)	27 (50.0%)	**<0.001**
Right	31 (31.0%)	11 (23.9%)	20 (37.0%)	
Bilateral	11 (11.0%)	11 (23.9%)	0 (0.0%)	
Unknown	18 (18.0%)	11 (23.9%)	7 (13.0%)	
Location (n, %)				
Renal cortex	5 (5.0%)	2 (4.3%)	3 (5.6%)	0.943
Renal sinus/hilus/medulla	35 (35.0%)	15 (32.6%)	20 (37.0%)	
Renal sinus/hilus/medulla and cortex	11 (11.0%)	5 (10.9%)	6 (11.1%)	
Unknown	49 (49.0%)	24 (52.2%)	25 (46.3%)	
Focality (n, %)				
Unifocal	61 (61.0%)	19 (41.3%)	42(77.8%)	**<0.001**
Multifocal	25 (25.0%)	23 (50.0%)	2 (3.7%)	
Unknown	14 (14.0%)	4 (8.7%)	10 (18.5%)	
Largest Tumor Diameter (mm) (n=81)	24.72 ± 22.31	16.79 ± 13.62	33.26 ± 26.51	**0.001**
Preoperative misdiagnosis (n=21)	21 (100.0%)	5 (23.8%)	16 (76.2%)	
Surgery approach (n, %) (n=91)				
Partial nephrectomy	20 (22.0%)	2 (4.5%)	18 (38.3%)	**<0.001**
Radical nephrectomy	71 (78.0%)	42 (95.5%)	29 (61.7%)	
Cooccurrence/History of other renal tumors (n=24)	24 (100.0%)	18 (75.0%)	6 (25.0%)	

Significant values are in bold.

#### 3.3.3 Comparison of RAHs and Renal Hemangiosarcoma

**Figure 16:**
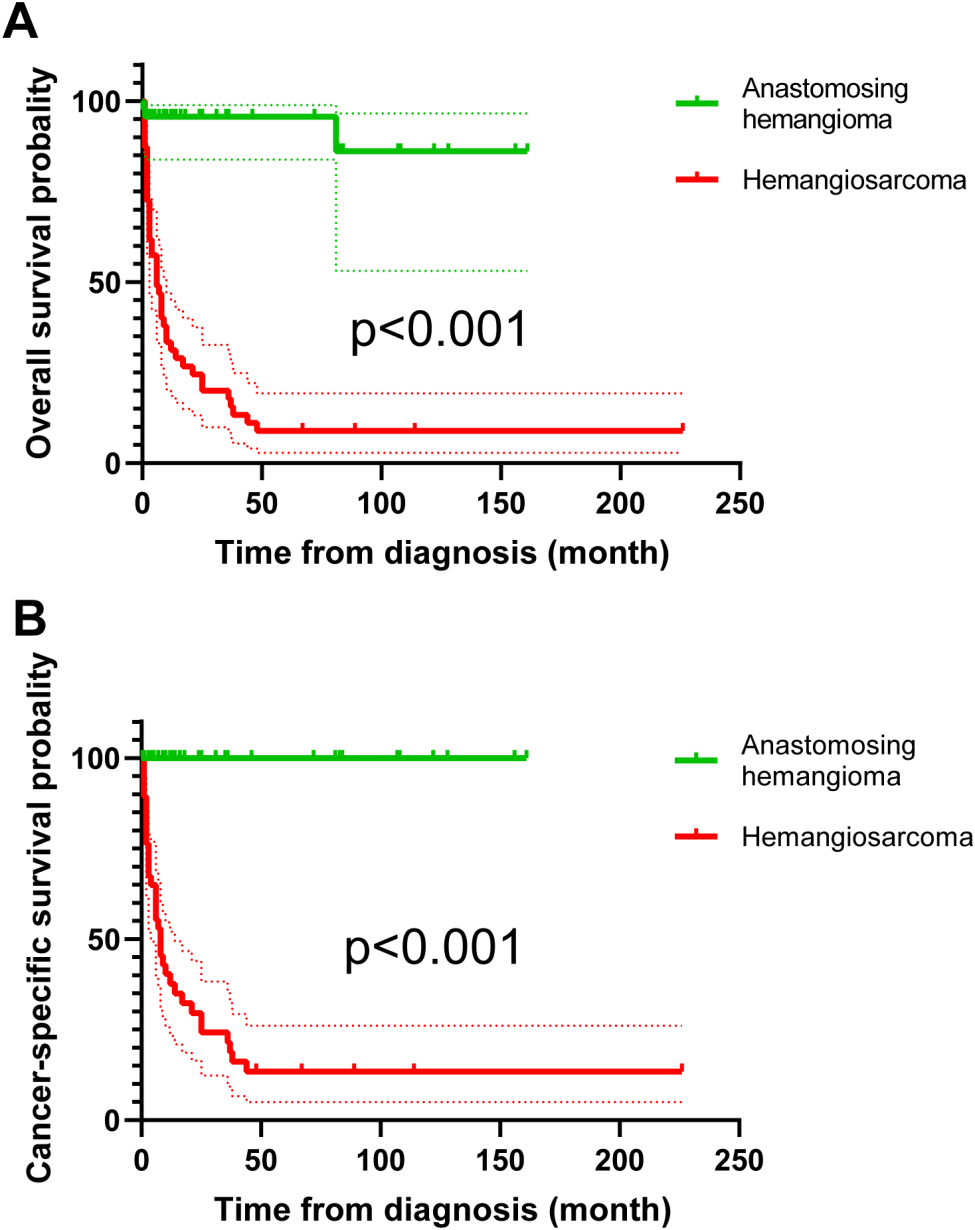
Survival analysis of overall survival **(A)** and cancer-specific survival **(B)** between renal anastomosing hemangioma and renal hemangiosarcoma patients.

In the comparison of clinical and tumor features between 100 RAH patients and 47 renal hemangiosarcoma patients, as shown in **Table 4**, RAH patients were diagnosed earlier than renal hemangiosarcoma patients were (*p*=0.001). The RAH tended to be bilateral at presentation (*p*<0.001) and to be associated with a smaller tumor size (*p*=0.001). A total of 47 RAH patients and 54 renal hemangiosarcoma patients with complete follow-up data were included in the survival analysis. As shown in **Figure 16**, the RAH patients exhibited significantly better OS and CSS than the renal hemangiosarcoma patients did (*p*<0.001).

**Table 4:** Comparison of clinical and tumor features between renal Anastomosing Hemangioma and Hemangiosarcoma.

	Anastomosing Hemangioma (n=100)	Hemangiosarcoma (n=53)	*p*
Age (Mean ± SD)	50.32 ± 16.96	63.00 ± 17.72	**0.001**
Gender (n, %)			
Female	32 (32.0%)	12 (22.6%)	0.224
Male	68 (68.0%)	41 (77.4%)	
Site (n, %)			
Left	40 (40.0%)	32 (60.4%)	**<0.001**
Right	31 (31.0%)	20 (37.7%)	
Bilateral	11 (11.0%)	0 (0.0%)	
Unknown	18 (18.0%)	1 (1.9%)	
Tumor size (mm, Mean ± SD)	24.72 ± 22.31	116.08 ± 53.12	**0.001**

Significant values are in bold.

## 4 Discussion

RAH is a rare benign subtype of benign hemangioma whose pathogenesis has not yet been fully elucidated. In our study, we present the case of a female patient with left RAH who underwent robotic-assisted laparoscopic partial left nephrectomy and remained in good condition with no signs of recurrence or metastasis after 27 months of regular follow-up. To clarify the genetic alterations and preliminarily explore the potential mechanism of the pathogenesis of RAH, we first applied WES to RAH and conducted a population-based study to comprehensively understand the clinicopathological characteristics of RAH.

There are two main accepted hypotheses for the pathogenesis of RAH. One is the ESRD-related mechanism, supported by RAH arising in ESRD patients as reported in publications from different institutions. Moreover, the greater percentage of bilateral (*p*=0.021) and multifocal (*p*<0.001) tumors in ESRD patients in our population-based study also implies a potential association between ESRD and RAH. ESRD is a fertile ground for malignant renal epithelial tumor development through the following proposed factors: the cellular and humoral immune deficiency caused by renal failure results in a decrease in antioxidant defenses, subsequent accumulation of reactive oxygen species, enhanced chronic infections and inflammation in association with phagocytic activity and increased free radical release, resulting in DNA damage, mutations and cancer progression. The accumulation of carcinogenic compounds, including carcinogenic heterocyclic amines, in ESRD and immunosuppressive medications can also impair methylation-dependent repair, which can inhibit the repair of DNA mutations and promote malignant cell transformation (27, 28). While some researchers hold different views, Kryvenko ON et al. suggested that the observed association between ESRD and AH was due to the bias that the surveillance imaging of ESRD patients promoted the detection of RAH (12). However, while this incidental association between ESRD and RAH has been proposed as an alternative hypothesis, the exact underlying mechanisms remain unknown and still need in-depth exploration.

A potential driving mechanism is that constitutive GTPase activity and the mitogen-activated protein (*MAP*) kinase signaling pathway are regulated by the recurrent mutant G protein alpha subunit *GNAQ* and its paralogs *GNA11* and *GNA14*, which are also expressed in other hemangiomas. *GNAQ* and *GNA11* also participate in vascular endothelial growth factor (*VEGF*) signaling and angiogenesis (13, 29, 30). Our study also detected mutant *GNAQ* mutations via WGS while didn’t confirm them via Sanger sequencing.

We identified mutations in the predisposing genes *CNTNAP2*, *NCOA2*, *FAT1*, *MET*, *TJP2*, *MAML2*, *SRGAP3*, and *CSMD3*, driver gene *HIP1* and validated them by Sanger sequencing. We also demonstrated that *FAT*, *MET* and *MAML2* mutations may be involved in the progression of RAH. *FAT1* (*FAT* atypical cadherin 1), which is a member of the *FAT* cadherin family in vertebrates, is a highly mutated gene in human cancers that encodes a protocadherin (31, 32). *FAT1* participated in multiple signaling pathways, including the *Wnt*/*β-catenin* signaling pathway, the Hippo signaling pathway, and the *MAPK*/*ERK* signaling pathway, and it mediates epithelial–mesenchymal transition to promote cell proliferation, migration and invasion (33–37). Owing to the importance of *FAT1* in organisms, mutations in *FAT1* may cause diverse malignant biological behaviors and have been detected in multiple diseases, such as acute lymphoblastic leukemia (38), hepatocellular carcinoma (39), pituitary spindle cell tumors (40), and head and neck squamous cell carcinoma (41, 42). Moreover, Zhou L et al. reported a mutation in *FAT1* in an acquired cystic disease-associated renal cell carcinoma that may have participated in its pathogenesis (43). The *FAT1* mutation we confirmed suggested a potential role for this mutation in the progression of RAH. *MET* is a proto-oncogene encoding the hepatocyte growth factor (HGF) tyrosine kinase receptor and it participates in regulating embryogenesis, angiogenesis, wound healing and liver regeneration (44). Alterations in *MET* can drive tumorigenesis through various molecular mechanisms in several types of cancer. Mutations in the tyrosine kinase domain (*TKD*) can lead to receptor phosphorylation and signaling unrelated to ligands. Mutations in this domain have been confirmed in type 1 papillary renal cell carcinomas and even cause resistance to *MET* tyrosine kinase inhibitors in lung cancers and neuroblastoma (44–47). Fang Y et al. reported that *HGF*/*Met* pathway deficiency caused by *MET* mutation impedes late thyroid expansion and subsequently leads to thyroid dysgenesis, and the *MET* mutation identified in the RAH in this study may be involved in the pathogenesis of innate developmental disorders (48). Mastermind-like transcriptional coactivator 2 (*MAML2*) is a member of the Mastermind-like family and is a transcriptional regulator of Notch signaling (49). The rearrangement of *MAML2* has been confirmed to be associated with a variety of diseases, such as atypical intraparenchymal meningioma (50) and metaplastic thymoma (51, 52). Linos K and Dermawan JK et al. also detected *MAML2* rearrangement in composite hemangioendotheliomas, which supports the function of *MAML2* in tumors originating from the vasculature (53, 54). The confirmed mutated genes and subsequent explorations may also contribute to the diagnosis of RAH and the development of targeted therapies in the future.

Another striking finding was that mutated genes may influence disease development by regulating the infiltration of immune cells, including NK/T cells, mast cells, M1 macrophages, M2 macrophages and plasmacytes, in the tumor microenvironment through the PPI analysis, the higher distribution of the tumor-associated macrophages (CD163) also validate the role in the disease. The regulation of macrophages in hemangioma has been thoroughly described in previous studies (55). M2 and M1 macrophages play almost opposite roles in infantile hemangioma, with M2 predominantly present in large numbers in the early stages of IH proliferation, promoting microvessel formation and lymphatic growth, whereas M1 macrophages exert antiproliferative and antitumor effects in the later stages of proliferation. Furthermore, the regulatory mechanisms of angiogenesis by which M1 macrophages contribute to regulating the proliferation and differentiation of hemangioma-derived stem cells and M2 macrophages facilitate the endothelial differentiation of hemangioma-derived stem cells, which also demonstrates the crucial role of immune cells in the molecular mechanisms and pathological processes of hemangioma (56, 57). Moreover, the identified combination of *CSMD3* and *MET* with PD-1 and two reported cases of hepatic cavernous hemangioma after treatment with PD-1 inhibitors suggest that PD-1 may regulate angiogenesis by modulating certain mechanisms (58).

However, because the clinical and radiographic features between RAH, RCC, and AS are highly similar and accurate preoperative diagnosis of RAH currently remain challenging, only a small minority of patients who undergo preoperative puncture biopsy can receive a definitive diagnosis on the basis of histology and immunochemistry. The routine radiographic examinations for renal masses include ultrasonography, CT and magnetic resonance imaging (MRI), but these methods still cannot distinguish RAH from RCC or angiosarcoma. These entities all present echogenicity on ultrasonography and a solid, boundary-cleared lesion with heterogeneous avid enhancement on contrast-enhanced CT (59). Nevertheless, RAH has some specific features on CT and MRI. On dynamic CT and MRI, RAH usually shows contrast enhancement from the periphery toward the center, with marked T2 hyperintensity similar to that of a cyst and persistent enhancement in the venous phase (7, 60). Although the RAH patient presented by Chua WM et al. underwent CT and ^68^Ga-DOTATATE PET/CT examinations, it was still misdiagnosed preoperatively as a neuroendocrine tumor (61). The differentiated diagnosis of RAH from AH in angiosarcoma patients with extensive hemorrhage described by Heo SH et al. was also confirmed by immunohistochemistry of operative samples rather than preoperative CT and MRI (62). Although the imaging findings are nonspecific and cannot further confirm the diagnosis of RAH, the above manifestations on imaging examinations, including CT, MRI and PET-CT, can also provide clues for radiologists and urologists to perform percutaneous renal biopsy to confirm the diagnosis to avoid nephrectomy. Notably, when invasive procedures, including percutaneous renal biopsy and ureteroscopy, are considered in patients with a suspected diagnosis of RAH, caution should be taken to prevent hemorrhage and spontaneous rupture of the RAH. The precise preoperative diagnosis of the RAH and its ability to differentiate it from other malignant renal tumors, such as renal cell carcinoma and angiosarcoma, could help patients choose more suitable treatment approaches, such as selective embolization and partial nephrectomy, rather than radical nephrectomy, and deep learning of radiographic images may serve useful purposes.

There are also several limitations of our study. Given the rare occurrence of RAH, more RAH samples are needed to verify the reliability and reproducibility of genetic alterations screened by WGS, and cell and animal experiments are also needed to validate the role of genetic alterations in RAH pathogenesis. Moreover, WGS of data from RAH patients with ESRD may contribute to further understanding of the mechanisms associated with ESRD. In our population-based study, we excluded some patients with RAH who lacked detailed information, which may have led to bias and inaccuracies. Moreover, owing to the limited number of RAH cases, the impact of disorders other than ESRD, including hypertension, type 2 diabetes, and pyelonephritis, on RAH has not been explored, and larger cohorts may contribute to clarification. Furthermore, we would also like to conduct deep learning using CT or MR images to distinguish the RAH from RCC and AS in the future.

## 5 Conclusion

RAH is a rare benign tumor, and the positive staining of markers, including ERG, FLI-1, CD31 and CD34, can contribute to its differential diagnosis. Pathologically, the expression content of tumor-associated macrophages and fibroblasts differs between cancerous and precancerous tissues. In the genomics field, mutations in predisposing genes, including *CNTNAP2*, *NCOA2*, *FAT1*, *MET*, *TJP2*, *MAML2*, *SRGAP3*, and *CSMD3* and driver gene *HIP1*, which were confirmed by WGS and verified by Sanger sequencing, may participate in the pathogenesis of RAH. However, further molecular biology experiments are still needed to explore the mechanism driving the development of RAH.

## Data Availability

The datasets presented in this study can be found in online repositories. The names of the repository/repositories and accession number(s) can be found below: https://www.ncbi.nlm.nih.gov/, SRR28364768 https://www.ncbi.nlm.nih.gov/, SRR28364767

## References

[1] Montgomery E Epstein JI. Anastomosing hemangioma of the genitourinary tract: a lesion mimicking angiosarcoma. Am J Surg Pathol. (2009) 33:1364–9. doi: 10.1097/PAS.0b013e3181ad30a7 19606014

[2] Humphrey PA Moch H Cubilla AL Ulbright TM Reuter VE. The 2016 WHO classification of tumours of the urinary system and male genital organs-part B: prostate and bladder tumours. Eur Urol. (2016) 70:106–19. doi: 10.1016/j.eururo.2016.02.028 26996659

[3] Omiyale AO. Clinicopathological features of primary angiosarcoma of the kidney: a review of 62 cases. Transl Androl Urol. (2015) 4(4):464–73. doi: 10.3978/j.issn.2223-4683.2015.05.04 PMC4708591 26816844

[4] Perdiki M Datseri G Liapis G Chondros N Anastasiou I Tzardi M. Anastomosing hemangioma: report of two renal cases and analysis of the literature. Diagn Pathol. (2017) 12:14. doi: 10.1186/s13000-017-0597-4 28118845 PMC5260082

[5] Omiyale AO. Anastomosing hemangioma of the kidney: a literature review of a rare morphological variant of hemangioma. Ann Transl Med. (2015) 3(11):151. doi: 10.3978/j.issn.2305-5839.2015.06.16 26244138 PMC4499659

[6] Katabathina VS Vikram R Nagar AM Tamboli P Menias CO Prasad SR. Mesenchymal neoplasms of the kidney in adults: imaging spectrum with radiologic-pathologic correlation. Radiographics. (2010) 30:1525–40. doi: 10.1148/rg.306105517 21071373

[7] Cheon PM Rebello R Naqvi A Popovic S Bonert M Kapoor A. Anastomosing hemangioma of the kidney: radiologic and pathologic distinctions of a kidney cancer mimic. Curr Oncol. (2018) 25:e220–3. doi: 10.3747/co.25.3927 PMC6023565 29962849

[8] Brown JG Folpe AL Rao P Lazar AJ Paner GP Gupta R. Primary vascular tumors and tumor-like lesions of the kidney: a clinicopathologic analysis of 25 cases. Am J Surg Pathol. (2010) 34:942–9. doi: 10.1097/PAS.0b013e3181e4f32a 20534992

[9] Kryvenko ON Gupta NS Meier FA Lee MW Epstein JI. Anastomosing hemangioma of the genitourinary system: eight cases in the kidney and ovary with immunohistochemical and ultrastructural analysis. Am J Clin Pathol. (2011) 136:450–7. doi: 10.1309/AJCPJPW34QCQYTMT 21846922

[10] Buttner M Kufer V Brunner K Hartmann A Amann K Agaimy A. Benign mesenchymal tumours and tumour-like lesions in end-stage renal disease. Histopathology. (2013) 62(2):229–36. doi: 10.1111/j.1365-2559.2012.04349.x 23020314

[11] Chou S Subramanian V Lau HM Achan A. Renal anastomosing hemangiomas with a diverse morphologic spectrum: report of two cases and review of literature. Int J Surg Pathol. (2014) 22:369–73. doi: 10.1177/1066896913492850 23816823

[12] Kryvenko ON Haley SL Smith SC Shen SS Paluru S Gupta NS. Haemangiomas in kidneys with end-stage renal disease: a novel clinicopathological association. Histopathology. (2014) 65:309–18. doi: 10.1111/his.2014.65.issue-3 24548339

[13] Bean GR Joseph NM Gill RM Folpe AL Horvai AE Umetsu SE. Recurrent GNAQ mutations in anastomosing hemangiomas. Mod Pathol. (2017) 30:722–7. doi: 10.1038/modpathol.2016.234 28084343

[14] Li H Durbin R. Fast and accurate long-read alignment with Burrows-Wheeler transform. Bioinformatics. (2010) 26:589–95. doi: 10.1093/bioinformatics/btp698 PMC2828108 20080505

[15] Li H Handsaker B Wysoker A Fennell T Ruan J Homer N. The sequence alignment/map format and SAMtools. Bioinformatics. (2009) 25:2078–9. doi: 10.1093/bioinformatics/btp352 PMC2723002 19505943

[16] Boeva V Popova T Bleakley K Chiche P Cappo J Schleiermacher G. Control-FREEC: a tool for assessing copy number and allelic content using next-generation sequencing data. Bioinformatics. (2012) 28:423–5. doi: 10.1093/bioinformatics/btr670 PMC3268243 22155870

[17] Layer RM Chiang C Quinlan AR Hall IM. LUMPY: a probabilistic framework for structural variant discovery. Genome Biol. (2014) 15:R84. doi: 10.1186/gb-2014-15-6-r84 24970577 PMC4197822

[18] Cibulskis K Lawrence MS Carter SL Sivachenko A Jaffe D Sougnez C. Sensitive detection of somatic point mutations in impure and heterogeneous cancer samples. Nat Biotechnol. (2013) 31:213–9. doi: 10.1038/nbt.2514 PMC3833702 23396013

[19] Saunders CT Wong WS Swamy S Becq J Murray LJ Cheetham RK. Strelka: accurate somatic small-variant calling from sequenced tumor-normal sample pairs. Bioinformatics. (2012) 28:1811–7. doi: 10.1093/bioinformatics/bts271 22581179

[20] Wang K Li M Hakonarson H. ANNOVAR: functional annotation of genetic variants from high-throughput sequencing data. Nucleic Acids Res. (2010) 38:e164. doi: 10.1093/nar/gkq603 20601685 PMC2938201

[21] Kandoth C McLellan MD Vandin F Ye K Niu B Lu C. Mutational landscape and significance across 12 major cancer types. Nature. (2013) 502:333–9. doi: 10.1038/nature12634 PMC3927368 24132290

[22] Tamborero D Gonzalez-Perez A Perez-Llamas C Deu-Pons J Kandoth C Reimand J. Comprehensive identification of mutational cancer driver genes across 12 tumor types. Sci Rep. (2013) 3:2650. doi: 10.1038/srep02650 24084849 PMC3788361

[23] Carter SL Cibulskis K Helman E McKenna A Shen H Zack T. Absolute quantification of somatic DNA alterations in human cancer. Nat Biotechnol. (2012) 30:413–21. doi: 10.1038/nbt.2203 PMC4383288 22544022

[24] Madaj R Geoffrey B Sanker A Valluri PP. Target2DeNovoDrug: a novel programmatic tool for in silico-deep learning based *de novo* drug design for any target of interest. J Biomol Struct Dyn. (2022) 40:7511–6. doi: 10.1080/07391102.2021.1898474 33703998

[25] Ashburner M Ball CA Blake JA Botstein D Butler H Cherry JM. Gene ontology: tool for the unification of biology. Gene Ontology Consortium Nat Genet. (2000) 25:25–9. doi: 10.1038/75556 PMC3037419 10802651

[26] Kanehisa M Goto S. KEGG: kyoto encyclopedia of genes and genomes. Nucleic Acids Res. (2000) 28:27–30. doi: 10.1093/nar/28.1.27 10592173 PMC102409

[27] Tickoo SK dePeralta-Venturina MN Harik LR Worcester HD Salama ME Young AN. Spectrum of epithelial neoplasms in end-stage renal disease: an experience from 66 tumor-bearing kidneys with emphasis on histologic patterns distinct from those in sporadic adult renal neoplasia. Am J Surg Pathol. (2006) 30:141–53. doi: 10.1097/01.pas.0000185382.80844.b1 16434887

[28] Truong LD Krishnan B Cao JT Barrios R Suki WN. Renal neoplasm in acquired cystic kidney disease. Am J Kidney Dis. (1995) 26:1–12. doi: 10.1016/0272-6386(95)90146-9 7611240

[29] Liau JY Tsai JH Lan J Chen CC Wang YH Lee JC. GNA11 joins GNAQ and GNA14 as a recurrently mutated gene in anastomosing hemangioma. Virchows Arch. (2020) 476:475–81. doi: 10.1007/s00428-019-02673-y 31707589

[30] Bean GR Joseph NM Folpe AL Horvai AE Umetsu SE. Recurrent GNA14 mutations in anastomosing haemangiomas. Histopathology. (2018) 73:354–7. doi: 10.1111/his.2018.73.issue-2 29574926

[31] Peng Z Gong Y Liang X. Role of FAT1 in health and disease. Oncol Lett. (2021) 21:398. doi: 10.3892/ol.2021.12659 33777221 PMC7988705

[32] Tanoue T Takeichi M. New insights into Fat cadherins. J Cell Sci. (2005) 118:2347–53. doi: 10.1242/jcs.02398 15923647

[33] Pastushenko I Mauri F Song Y de Cock F Meeusen B Swedlund B. Fat1 deletion promotes hybrid EMT state, tumour stemness and metastasis. Nature. (2021) 589:448–55. doi: 10.1038/s41586-020-03046-1 PMC7612440 33328637

[34] Hu X Zhai Y Kong P Cui H Yan T Yang J. FAT1 prevents epithelial mesenchymal transition (EMT) via MAPK/ERK signaling pathway in esophageal squamous cell cancer. Cancer Lett. (2017) 397:83–93. doi: 10.1016/j.canlet.2017.03.033 28366557

[35] Ahmed AF de Bock CE Lincz LF Pundavela J Zouikr I Sontag E. FAT1 cadherin acts upstream of Hippo signalling through TAZ to regulate neuronal differentiation. Cell Mol Life Sci. (2015) 72:4653–69. doi: 10.1007/s00018-015-1955-6 PMC11113810 26104008

[36] Morris LG Kaufman AM Gong Y Ramaswami D Walsh LA Turcan S. Recurrent somatic mutation of FAT1 in multiple human cancers leads to aberrant Wnt activation. Nat Genet. (2013) 45:253–61. doi: 10.1038/ng.2538 PMC3729040 23354438

[37] Schreiner D Muller K Hofer HW. The intracellular domain of the human protocadherin hFat1 interacts with Homer signalling scaffolding proteins. FEBS Lett. (2006) 580:5295–300. doi: 10.1016/j.febslet.2006.08.079 16979624

[38] Dunne J Hanby AM Poulsom R Jones TA Sheer D Chin WG. Molecular cloning and tissue expression of FAT, the human homologue of the Drosophila fat gene that is located on chromosome 4q34-q35 and encodes a putative adhesion molecule. Genomics. (1995) 30:207–23. doi: 10.1006/geno.1995.9884 8586420

[39] Valletta D Czech B Spruss T Ikenberg K Wild P Hartmann A. Regulation and function of the atypical cadherin FAT1 in hepatocellular carcinoma. Carcinogenesis. (2014) 35:1407–15. doi: 10.1093/carcin/bgu054 24590895

[40] Miller MB Bi WL Ramkissoon LA Kang YJ Abedalthagafi M Knoff DS. MAPK activation and HRAS mutation identified in pituitary spindle cell oncocytoma. Oncotarget. (2016) 7:37054–63. doi: 10.18632/oncotarget.v7i24 PMC5095058 27175596

[41] Lin SC Lin LH Yu SY Kao SY Chang KW Cheng HW. FAT1 somatic mutations in head and neck carcinoma are associated with tumor progression and survival. Carcinogenesis. (2018) 39:1320–30. doi: 10.1093/carcin/bgy107 30102337

[42] Katoh Y Katoh M. Comparative integromics on FAT1, FAT2, FAT3 and FAT4. Int J Mol Med. (2006) 18:523–8. doi: 10.3892/ijmm.18.3.523 16865240

[43] Zhou L Xu H Liu Y Li X Li C Yang X. Acquired cystic disease-associated renal cell carcinoma with PTCH1 mutation: a case report. Front Oncol. (2024) 14:1349610. doi: 10.3389/fonc.2024.1349610 38371617 PMC10870146

[44] Recondo G Che J Janne PA Awad MM. Targeting MET dysregulation in cancer. Cancer Discov. (2020) 10:922–34. doi: 10.1158/2159-8290.CD-19-1446 PMC7781009 32532746

[45] Rotow JK Gui P Wu W Raymond VM Lanman RB Kaye FJ. Co-occurring alterations in the RAS-MAPK pathway limit response to MET inhibitor treatment in MET exon 14 skipping mutation-positive lung cancer. Clin Cancer Res. (2020) 26:439–49. doi: 10.1158/1078-0432.CCR-19-1667 PMC6980768 31548343

[46] Cancer Genome Atlas Research N Linehan WM Spellman PT Ricketts CJ Creighton CJ Fei SS. Comprehensive molecular characterization of papillary renal-cell carcinoma. N Engl J Med. (2016) 374:135–45. doi: 10.1056/NEJMoa1505917 PMC4775252 26536169

[47] Chen Y Takita J Choi YL Kato M Ohira M Sanada M. Oncogenic mutations of ALK kinase in neuroblastoma. Nature. (2008) 455:971–4. doi: 10.1038/nature07399 18923524

[48] Fang Y Wan JP Wang Z Song SY Zhang CX Yang L. Deficiency of the HGF/Met pathway leads to thyroid dysgenesis by impeding late thyroid expansion. Nat Commun. (2024) 15:3165. doi: 10.1038/s41467-024-47363-9 38605010 PMC11009301

[49] Lindberg MJ Popko-Scibor AE Hansson ML Wallberg AE. SUMO modification regulates the transcriptional activity of MAML1. FASEB J. (2010) 24:2396–404. doi: 10.1096/fj.09-149401 20203086

[50] Nobee A Xu M Seth A Rong Y. Atypical intraparenchymal meningioma with YAP1-MAML2 fusion in a young adult male: A case report and mini literature review. Int J Mol Sci. (2023) 24(16):12814. doi: 10.3390/ijms241612814 37628996 PMC10454436

[51] Wang X Liu LL Li Q Xia QY Li R Ye SB. Loss of YAP1 C-terminus expression as an ancillary marker for metaplastic thymoma: a potential pitfall in detecting YAP1::MAML2 gene rearrangement. Histopathology. (2023) 83:798–809. doi: 10.1111/his.15024 37565303

[52] Brownstein MH Rabinowitz AD. The invisible dermatoses. J Am Acad Dermatol. (1983) 8:579–88. doi: 10.1016/S0190-9622(83)80078-4 6853792

[53] Linos K Dermawan JK Pulitzer M Hameed M Agaram NP Agaimy A. Untying the Gordian knot of composite hemangioendothelioma: Discovery of novel fusions. Genes Chromosomes Cancer. (2024) 63:e23198. doi: 10.1002/gcc.23198 37658696 PMC10842102

[54] Dermawan JK Westra WH Antonescu CR. Recurrent PTBP1::MAML2 fusions in composite hemangioendothelioma with neuroendocrine differentiation: A report of two cases involving neck lymph nodes. Genes Chromosomes Cancer. (2022) 61:187–93. doi: 10.1002/gcc.23017 PMC8895320 34862698

[55] Xiang S Gong X Qiu T Zhou J Yang K Lan Y. Insights into the mechanisms of angiogenesis in infantile hemangioma. Biomedicine pharmacotherapy = Biomedecine pharmacotherapie. (2024) 178:117181. doi: 10.1016/j.biopha.2024.117181 39059349

[56] Wu KQ Muratore CS So EY Sun C Dubielecka PM Reginato AM. M1 macrophage-induced endothelial-to-mesenchymal transition promotes infantile hemangioma regression. Am J Pathol. (2017) 187:2102–11. doi: 10.1016/j.ajpath.2017.05.014 PMC5809337 28710904

[57] Zhang W Chen G Wang FQ Ren JG Zhu JY Cai Y. Macrophages contribute to the progression of infantile hemangioma by regulating the proliferation and differentiation of hemangioma stem cells. J Invest Dermatol. (2015) 135:3163–72. doi: 10.1038/jid.2015.321 26288359

[58] Finlay WJJ Coleman JE Edwards JS Johnson KS. Anti-PD1 ‘SHR-1210’ aberrantly targets pro-angiogenic receptors and this polyspecificity can be ablated by paratope refinement. mAbs. (2019) 11(1):26–44. doi: 10.1080/19420862.2018.1550321 30541416 PMC6343799

[59] Lee HS Koh BH Kim JW Kim YS Rhim HC Cho OK. Radiologic findings of renal hemangioma: report of three cases. Korean J Radiol. (2000) 1:60–3. doi: 10.3348/kjr.2000.1.1.60 PMC2718141 11752931

[60] Capinha MD Carvalho-Dias E Cerqueira-Alves M Mota P. Renal anastomosing haemangioma. BMJ Case Rep. (2023) 16(9):e254131. doi: 10.1136/bcr-2022-254131 PMC10510885 37723090

[61] Chua WM Hoe K Dalan R Too CW Ong S Tay T. Anastomosing hemangioma on 68Ga-DOTATATE PET/CT: A potential pitfall. Clin Nucl Med. (2022) 47(4):321–3. doi: 10.1097/RLU.0000000000003984 35020655

[62] Heo SH Shin SS Kang TW Kim GE. Primary renal angiosarcoma with extensive hemorrhage: CT and MRI findings. Int Braz J Urol. (2019) 45:402–5. doi: 10.1590/s1677-5538.ibju.2018.0375 PMC6541118 30735338

