# Supplementary material for: Clinicopathological features and genetic mutation spectrum of primary anastomosing hemangioma arising from the kidney
Source: Front Immunol. 2025 May 9;16:1554203. doi: 10.3389/fimmu.2025.1554203 (PMC12098607; doi:10.3389/fimmu.2025.1554203)

**Supplementary materials**

**Supplementary tables**

**Table S1**. Antibodies used in the immunohistochemistry. Ig, Immunoglobulin; CD, cluster of differentiation.

| **Antibody** | **Species** | **Antibody number** | **Source** | **Dilution** |
| --- | --- | --- | --- | --- |
| CD31 | Rabbit polyclonal | ab28364 | Abcam, Cambridge, UK | 1:50 |
| CD34 | Rabbit monoclonal | ab110643 | Abcam, Cambridge, UK | 1:50 |
| SMA | Mouse monoclonal | MA1-06110 | ThermoFisher Scientific, Massachusetts, USA | 1:200 |
| D2-40  (Podoplanin) | Mouse monoclonal | MA5-16113 | ThermoFisher Scientific, Massachusetts, USA | 1:400 |
| EMA  (MUC1) | Mouse monoclonal | MA5- 11202 | ThermoFisher Scientific, Massachusetts, USA | 1:200 |
| AE1/AE3 | Mouse monoclonal | A500- 019A | ThermoFisher Scientific, Massachusetts, USA | 1:200 |
| HMB-45  (Melanoma gp10) | Mouse monoclonal | ab787 | Abcam, Cambridge, UK | 1:200 |
| Ki-67 | Rabbit polyclonal | 27309-1-AP | Protein Tech Group, Chicago, USA | 1:6000 |
| FL1-1 | Rabbit polyclonal | PA5-29597 | ThermoFisher Scientific, Massachusetts, USA | 1:500 |
| ERG | Mouse monoclonal | MA5-26261 | ThermoFisher Scientific, Massachusetts, USA | 1:50 |
| GLUT-1 | Rabbit monoclonal | ab115730 | Abcam, Cambridge, UK | 1:400 |
| PD-L1 | Rabbit monoclonal | ab205921 | Abcam, Cambridge, UK | 2 μg ml^−1^ |
| CD163 | Rabbit monoclonal | ab182422 | Abcam, Cambridge, UK | 1: 400 |
| CD68 | Mouse monoclonal | ab955 | Abcam, Cambridge, UK | 1: 3000 |

**Table S2**. The number of SNPs on different regions of the genome and coding regions. CDS, coding sequences; SNP, single nucleotide polymorphism; UTR, untranslated region; ncRNA, noncoding ribonucleic acid.

| **Sample** | **Normal specimen** | **Tumor specimen** |
| --- | --- | --- |
| CDS | 22662 | 22447 |
| Synonymous_SNP | 11542 | 11447 |
| Missense_SNP | 10506 | 10387 |
| Stopgain | 88 | 85 |
| Stoploss | 14 | 14 |
| Unknown | 487 | 491 |
| Intronic | 1302978 | 1290485 |
| UTR3 | 33438 | 33163 |
| UTR5 | 6351 | 6284 |
| Splicing | 645 | 644 |
| ncRNA_exonic | 14861 | 14704 |
| ncRNA_intronic | 240180 | 237069 |
| ncRNA_UTR3 | 0 | 0 |
| ncRNA_UTR5 | 0 | 0 |
| ncRNA_splicing | 175 | 171 |
| Upstream | 23360 | 23013 |
| Downstream | 24589 | 24294 |
| Intergenic | 2009645 | 1985155 |
| Total | 3678071 | 3636612 |

**Table S3.** The number of SNPs in different regions of the genome. SNP, single nucleotide polymorphism; Het, heterozygote; Hom, homozygote; TS, transformation; TV, transmutation; dbSNP, the single nucleotide polymorphism.

| **Sample** | **Normal specimen** | **Tumor specimen** |
| --- | --- | --- |
| Total | 3678071 | 3636612 |
| Het | 2147255 | 2121038 |
| Hom | 1530816 | 1515574 |
| TS | 2469501 | 2444756 |
| TV | 1208570 | 1191856 |
| TS/TV | 2.04 | 2.05 |
| dbSNP percentage | 3653337(99.33%) | 3612300(99.33%) |
| Novel | 24734 | 24312 |
| Novel_ TS | 14783 | 14653 |
| Novel_TV | 9951 | 9659 |
| Novel_TS/TV | 1.49 | 1.52 |

**Table S4.** The number of INDELs on different regions of the genome and coding regions. CDS, coding sequences; SNP, single nucleotide polymorphism; UTR, untranslated region; ncRNA, noncoding ribonucleic acid.

| **Sample** | **Normal specimen** | **Tumor specimen** |
| --- | --- | --- |
| CDS | 591 | 594 |
| Frameshift deletion | 60 | 61 |
| Frameshift insertion | 62 | 61 |
| Nonframeshift deletion | 183 | 194 |
| Nonframeshift insertion | 175 | 166 |
| Stopgain | 10 | 8 |
| Stoploss | 0 | 3 |
| Unknown | 99 | 99 |
| Intronic | 419490 | 418374 |
| UTR3 | 11565 | 11527 |
| UTR5 | 1198 | 1170 |
| Splicing | 402 | 424 |
| ncRNA_exonic | 2384 | 2347 |
| ncRNA_intronic | 68130 | 67213 |
| ncRNA_UTR3 | 0 | 0 |
| ncRNA_UTR5 | 2 | 3 |
| ncRNA_splicing | 98 | 90 |
| Upstream | 7435 | 7188 |
| Downstream | 8533 | 8492 |
| Intergenic | 542110 | 536063 |
| Total | 1061622 | 1053180 |

**Table S5.** The number of INDELs in different regions of the genome. INDELs, insertions and deletions; Het, heterozygote; Hom, homozygote; dbSNP, the single nucleotide polymorphism.

| **Sample** | **Normal specimen** | **Tumor specimen** |
| --- | --- | --- |
| Total | 1061622 | 1053180 |
| Het | 831275 | 827358 |
| Hom | 230347 | 225822 |
| dbSNP percentage | 859410(80.95%) | 841717(79.92%) |
| Novel | 202212 | 211463 |
| Novel_proportion | 19.05% | 20.08% |

**Table S6.** The number of Somatic SNVs in different regions of the genome. SNVs, single nucleotide variants; CDS, coding sequence; SNP, single nucleotide polymorphism; UTR, untranslated region; ncRNA, noncoding ribonucleic acid.

| **Sample** | **Tumor specimen** |
| --- | --- |
| CDS | 119 |
| synonymous_SNP | 36 |
| missense_SNP | 73 |
| stopgain | 7 |
| stoploss | 0 |
| unknown | 3 |
| intronic | 3816 |
| UTR3 | 99 |
| UTR5 | 17 |
| splicing | 1 |
| ncRNA_exonic | 50 |
| ncRNA_intronic | 808 |
| ncRNA_UTR3 | 0 |
| ncRNA_UTR5 | 0 |
| ncRNA_splicing | 0 |
| upstream | 71 |
| downstream | 68 |
| intergenic | 5482 |
| Others | 1 |
| Total | 10532 |

**Table S7.** The number of Somatic INDELs in different regions of the genome. INDELs, insertions and deletions; ncRNA, noncoding ribonucleic acid.

| **Sample** | **Tumor specimen** |
| --- | --- |
| CDS | 31 |
| frameshift_deletion | 21 |
| frameshift_insertion | 2 |
| nonframeshift_deletion | 7 |
| nonframeshift_insertion | 0 |
| stopgain | 0 |
| stoploss | 1 |
| unknown | 0 |
| intronic | 2653 |
| UTR3 | 100 |
| UTR5 | 15 |
| splicing | 1 |
| ncRNA_exonic | 12 |
| ncRNA_intronic | 436 |
| ncRNA_UTR3 | 0 |
| ncRNA_UTR5 | 0 |
| ncRNA_splicing | 1 |
| upstream | 38 |
| downstream | 53 |
| intergenic | 3362 |
| Others | 3 |
| Total | 6705 |

**Table S8.** Analysis results of predisposing genes. Ref, reference; Alt, alternative; AA, amino acid.

| **Hugo**  **Symbol** | **Chromosome** | **Position** | **Ref**  **allele** | **Alt**  **allele** | **Variant_Classification** | **AAChange** |
| --- | --- | --- | --- | --- | --- | --- |
| PTPRT | 20 | 40710573 | G | C | Missense_Mutation | PTPRT:NM_007050:exon30:c.C4221G:p.F1407L  PTPRT:NM_133170:exon31:c.C4278G:p.F1426L |
| TNFAIP3 | 6 | 138196868 | C | T | Missense_Mutation | TNFAIP3:NM_001270507:exon4:c.C530T:p.T177I  TNFAIP3:NM_001270508:exon4:c.C530T:p.T177I  TNFAIP3:NM_006290:exon4:c.C530T:p.T177I |
| CNTNAP2 | 7 | 146818068 | C | T | Splice_Site | NM_014141:exon5:c.658-3C>T |
| CANT1 | 17 | 76989939 | C | T | Missense_Mutation | CANT1:NM_138793:exon4:c.G899A:p.R300H  CANT1:NM_001159773:exon5:c.G899A:p.R300H  CANT1:NM_001159772:exon6:c.G899A:p.R300H |
| CTNNA2 | 2 | 80085156 | G | A | Missense_Mutation | CTNNA2:NM_001164883:exon4:c.G316A:p.A106T  CTNNA2:NM_001282597:exon4:c.G316A:p.A106T  CTNNA2:NM_001282598:exon4:c.G418A:p.A140T  CTNNA2:NM_004389:exon4:c.G316A:p.A106T |
| NCOA2 | 8 | 71033538 | C | T | Missense_Mutation | NCOA2:NM_001321711:exon21:c.G4175A:p.R1392Q  NCOA2:NM_001321712:exon21:c.G4175A:p.R1392Q  NCOA2:NM_001321713:exon21:c.G3920A:p.R1307Q  NCOA2:NM_001321703:exon22:c.G4382A:p.R1461Q  NCOA2:NM_001321707:exon22:c.G4382A:p.R1461Q  NCOA2:NM_006540:exon22:c.G4382A:p.R1461Q |
| FAT1 | 4 | 187539340 | T | G | Missense_Mutation | FAT1:NM_005245:exon10:c.A8400C:p.K2800N |
| MET | 7 | 116340087 | C | T | Missense_Mutation | MET:NM_000245:exon2:c.C949T:p.L317F  MET:NM_001127500:exon2:c.C949T:p.L317F  MET:NM_001324401:exon2:c.C949T:p.L317F |
| TJP2 | 9 | 71851954 | G | A | Missense_Mutation | TJP2:NM_001170415:exon14:c.G2093A:p.G698E  TJP2:NM_001170416:exon14:c.G2174A:p.G725E  TJP2:NM_001369872:exon14:c.G2081A:p.G694E  TJP2:NM_001369873:exon14:c.G2081A:p.G694E  TJP2:NM_001369874:exon14:c.G2093A:p.G698E  TJP2:NM_001369875:exon14:c.G2093A:p.G698E  TJP2:NM_004817:exon14:c.G2081A:p.G694E  TJP2:NM_201629:exon14:c.G2081A:p.G694E  TJP2:NM_001170414:exon15:c.G2012A:p.G671E  TJP2:NM_001369870:exon15:c.G2006A:p.G669E  TJP2:NM_001369871:exon16:c.G2012A:p.G671E |
| PCSK5 | 9 | 78936363 | T | C | Missense_Mutation | PCSK5:NM_001190482:exon30:c.T3829C:p.C1277R  PCSK5:NM_001372043:exon31:c.T3910C:p.C1304R |
| APC | 5 | 112175640 | G | C | Missense_Mutation | APC:NM_001127511:exon14:c.G4295C:p.R1432P  APC:NM_001354897:exon15:c.G4379C:p.R1460P  APC:NM_001354899:exon15:c.G4265C:p.R1422P  APC:NM_001354901:exon15:c.G4172C:p.R1391P  APC:NM_001354902:exon15:c.G4076C:p.R1359P  APC:NM_001354905:exon15:c.G3869C:p.R1290P  APC:NM_000038:exon16:c.G4349C:p.R1450P  APC:NM_001354895:exon16:c.G4349C:p.R1450P  APC:NM_001354898:exon16:c.G4274C:p.R1425P  APC:NM_001354900:exon16:c.G4226C:p.R1409P  APC:NM_001354903:exon16:c.G4046C:p.R1349P  APC:NM_001354904:exon16:c.G3971C:p.R1324P  APC:NM_001127510:exon17:c.G4349C:p.R1450P  APC:NM_001354896:exon17:c.G4403C:p.R1468P  APC:NM_001354906:exon17:c.G3500C:p.R1167P |
| MAML2 | 11 | 96074675 | C | T | Missense_Mutation | MAML2:NM_032427:exon1:c.G385A:p.D129N |
| PSIP1 | 9 | 15474122 | G | A | Missense_Mutation | PSIP1:NM_021144:exon8:c.C743T:p.P248L  PSIP1:NM_001128217:exon9:c.C743T:p.P248L  PSIP1:NM_001317898:exon9:c.C743T:p.P248L  PSIP1:NM_001317900:exon9:c.C716T:p.P239L  PSIP1:NM_033222:exon9:c.C743T:p.P248L |
| SRGAP3 | 3 | 9121722 | T | C | Missense_Mutation | SRGAP3:NM_001033117:exon4:c.A473G:p.N158S  SRGAP3:NM_014850:exon4:c.A473G:p.N158S |
| ZFHX3 | 16 | 72821730 | C | A | Missense_Mutation | ZFHX3:NM_001164766:exon9:c.G7703T:p.S2568I  ZFHX3:NM_006885:exon10:c.G10445T:p.S3482I |
| POLE | 12 | 133237641 | C | T | Missense_Mutation | POLE:NM_006231:exon25:c.G2974A:p.A992T |
| CSMD3 | 8 | 113347624 | T | C | Missense_Mutation | CSMD3:NM_001363185:exon41:c.A6499G:p.N2167D  CSMD3:NM_052900:exon44:c.A6787G:p.N2263D  CSMD3:NM_198123:exon45:c.A7099G:p.N2367D  CSMD3:NM_198124:exon46:c.A6979G:p.N2327D |
| MYH14 | 19 | 50774700 | C | T | Missense_Mutation | MYH14:NM_024729:exon23:c.C2945T:p.A982V  MYH14:NM_001077186:exon24:c.C2969T:p.A990V  MYH14:NM_001145809:exon25:c.C3068T:p.A1023V |
| DHX9 | 1 | 182821420 | AAGG | A | In_Frame_Del | DHX9:NM_001357:exon4:c.306_308del:p.G103del |
| MNX1 | 7 | 156802648 | C | CGGCGGG | In_Frame_Ins | MNX1:NM_005515:exon1:c.396_397insCCCGCC:p.A132_A133insPA |
| AR | X | 66766356 | TGGC | T | In_Frame_Del | AR:NM_000044:exon1:c.1369_1371del:p.G473del  AR:NM_001348061:exon1:c.1369_1371del:p.G473del  AR:NM_001348063:exon1:c.1369_1371del:p.G473del  AR:NM_001348064:exon1:c.1369_1371del:p.G473del |
| MNX1 | 7 | 156802643 | A | AGCGGCGGCG | In_Frame_Ins | MNX1:NM_005515:exon1:c.401_402insCGCCGCCGC:p.A134_G135insAAA |
| RABEP1 | 17 | 5286861 | GTAGTGTTTGGAATTTTCTGTTCATA | G | Splice_Site | . |
| MNX1 | 7 | 156802641 | C | CCCGCGCCGG | In_Frame_Ins | MNX1:NM_005515:exon1:c.403_404insCCGGCGCGG:p.A134_G135insAGA |
| RB1 | 13 | 48996928 | TA | T | Splice_Site | NM_001162497:exon2:UTR5  NM_001377316:exon2:UTR5  NM_001377317:exon2:UTR5  NM_005767:exon3:UTR5 |
| MSH2 | 2 | 47641559 | TAA | T | Splice_Site | NM_000251:exon5:r.spl  NM_001258281:exon6:r.spl |
| PBRM1 | 3 | 52676064 | A | AT | Splice_Site | NM_001350074:exon13:c.1059-3->A  NM_001350075:exon11:c.996-3->A  NM_001350076:exon13:c.1059-3->A  NM_001350077:exon14:c.1050-3->A  NM_001350078:exon13:c.1059-3->A  NM_001350079:exon13:c.1059-3->A  NM_001366070:exon12:c.1059-3->A  NM_001366071:exon12:c.1059-3->A  NM_001366072:exon12:c.1059-3->A  NM_001366073:exon12:c.1050-3->A  NM_001366074:exon13:c.1047-3->A  NM_001366075:exon12:c.1059-3->A  NM_001366076:exon11:c.954-3->A  NM_018313:exon11:c.996-3->A  NM_181042:exon12:c.996-3->A |
| TSC1 | 9 | 135773000 | G | GAA | Splice_Site | NM_000368:exon21:c.2626-3->TT  NM_001162426:exon21:c.2623-3->TT  NM_001162427:exon20:c.2473-3->TT  NM_001362177:exon20:c.2263-3->TT |
| BRAF | 7 | 140434574 | C | CA | Splice_Site | NM_001354609:exon18:c.2128-4->T  NM_001374244:exon19:c.2248-4->T  NM_001374258:exon19:c.2248-4->T  NM_001378467:exon18:c.2137-4->T  NM_001378469:exon18:c.2062-4->T  NM_001378470:exon17:c.2026-4->T  NM_001378471:exon17:c.2017-4->T  NM_001378472:exon18:c.1972-4->T  NM_001378473:exon18:c.1972-4->T  NM_001378475:exon17:c.1864-4->T  NM_004333:exon18:c.2128-4->T |
| POLE | 12 | 133237753 | GA | G | Splice_Site | NM_006231:exon25:c.2865-4T>- |
| AR | X | 66765158 | T | TGCA | In_Frame_Ins | AR:NM_000044:exon1:c.170_171insGCA:p.Q80_E81insQ  AR:NM_001348061:exon1:c.170_171insGCA:p.Q80_E81insQ  AR:NM_001348063:exon1:c.170_171insGCA:p.Q80_E81insQ  AR:NM_001348064:exon1:c.170_171insGCA:p.Q80_E81insQ |
| PTPRT | 20 | 40710573 | G | C | Missense_Mutation | PTPRT:NM_007050:exon30:c.C4221G:p.F1407L  PTPRT:NM_133170:exon31:c.C4278G:p.F1426L |
| TNFAIP3 | 6 | 138196868 | C | T | Missense_Mutation | TNFAIP3:NM_001270507:exon4:c.C530T:p.T177I  TNFAIP3:NM_001270508:exon4:c.C530T:p.T177I  TNFAIP3:NM_006290:exon4:c.C530T:p.T177I |
| CNTNAP2 | 7 | 146818068 | C | T | Splice_Site | NM_014141:exon5:c.658-3C>T |

**Table S9.** Analysis results of driving genes. Ref, reference; Alt, alternative; AA, amino acid.

| **Hugo**  **Symbol** | **Chromosome** | **Position** | **Ref**  **allele** | **Alt**  **allele** | **Variant_Classification** | **AAChange** |
| --- | --- | --- | --- | --- | --- | --- |
| CHD3 | 17 | 7801827 | G | A | Missense_Mutation | CHD3:NM_001005271:exon13:c.G2242A:p.G748R\|CHD3:NM_001005273:exon13:c.G2065A:p.G689R\|CHD3:NM_005852:exon13:c.G2065A:p.G689R |
| PRKCB | 16 | 23999906 | T | G | Missense_Mutation | PRKCB:NM_002738:exon3:c.T283G:p.S95A\|PRKCB:NM_212535:exon3:c.T283G:p.S95A |
| SPEG | 2 | 220337743 | C | T | Missense_Mutation | SPEG:NM_005876:exon16:c.C4072T:p.R1358W |
| CHD7 | 8 | 61655100 | G | A | Missense_Mutation | CHD7:NM_001316690:exon1:c.G1109A:p.S370N\|CHD7:NM_017780:exon2:c.G1109A:p.S370N |
| KAT6B | 10 | 76735809 | C | T | Missense_Mutation | KAT6B:NM_001370136:exon8:c.C1714T:p.R572C\|KAT6B:NM_001370137:exon8:c.C1714T:p.R572C\|KAT6B:NM_012330:exon8:c.C1714T:p.R572C |
| HIP1 | 7 | 75186080 | C | A | Missense_Mutation | HIP1:NM_001243198:exon17:c.G1617T:p.K539N\|HIP1:NM_001382444:exon17:c.G1515T:p.K505N\|HIP1:NM_001382445:exon17:c.G1530T:p.K510N\|HIP1:NM_005338:exon17:c.G1617T:p.K539N |
| ERBB4 | 2 | 212530069 | C | T | Missense_Mutation | ERBB4:NM_001042599:exon15:c.G1850A:p.C617Y\|ERBB4:NM_005235:exon15:c.G1850A:p.C617Y |
| RUNX1T1 | 8 | 93026809 | C | T | Missense_Mutation | RUNX1T1:NM_175636:exon2:c.G355A:p.V119M\|RUNX1T1:NM_001198633:exon3:c.G406A:p.V136M\|RUNX1T1:NM_001198634:exon3:c.G499A:p.V167M\|RUNX1T1:NM_004349:exon3:c.G385A:p.V129M\|RUNX1T1:NM_175635:exon3:c.G355A:p.V119M\|RUNX1T1:NM_001198628:exon4:c.G466A:p.V156M\|RUNX1T1:NM_001198629:exon4:c.G466A:p.V156M\|RUNX1T1:NM_001198631:exon4:c.G466A:p.V156M\|RUNX1T1:NM_001198632:exon4:c.G385A:p.V129M\|RUNX1T1:NM_001198679:exon4:c.G643A:p.V215M\|RUNX1T1:NM_175634:exon4:c.G466A:p.V156M\|RUNX1T1:NM_001198626:exon5:c.G466A:p.V156M\|RUNX1T1:NM_001198630:exon5:c.G466A:p.V156M\|RUNX1T1:NM_001198627:exon6:c.G466A:p.V156M\|RUNX1T1:NM_001198625:exon7:c.G385A:p.V129M |
| NIPBL | 5 | 37063924 | TGAA | T | In_Frame_Del | NIPBL:NM_015384:exon46:c.7894_7896del:p.E2636del\|NIPBL:NM_133433:exon46:c.7894_7896del:p.E2636del |
| CHD3 | 17 | 7788145 | CGAG | C | In_Frame_Del | CHD3:NM_001005271:exon1:c.22_24del:p.E14del |

**Table S10.** Prediction of targeted drugs based on somatic mutations. Chr, chromosome; AA, amino acid; N/A, not applicable.

| Gene symbol | Entriz id | Chr | Start position | End position | Variant Classification | AA Change | Drug name | Drug type | Interaction type | Source |
| --- | --- | --- | --- | --- | --- | --- | --- | --- | --- | --- |
| *AR* | 367 | X | 66766355 | 66766355 | Missense_Mutation | *AR*:NM_000044:exon1:c.G1367A:p.G456D | levonorgestrel | small molecule | agonist | DrugBank |
| *AR* | 367 | X | 66766355 | 66766355 | Missense_Mutation | *AR*:NM_000044:exon1:c.G1367A:p.G456D | spironolactone | small molecule | antagonist | DrugBank |
| *AR* | 367 | X | 66766355 | 66766355 | Missense_Mutation | *AR*:NM_000044:exon1:c.G1367A:p.G456D | flutamide | small molecule | antagonist | DrugBank |
| *AR* | 367 | X | 66766355 | 66766355 | Missense_Mutation | *AR*:NM_000044:exon1:c.G1367A:p.G456D | oxandrolone | small molecule | agonist | DrugBank |
| *AR* | 367 | X | 66766355 | 66766355 | Missense_Mutation | *AR*:NM_000044:exon1:c.G1367A:p.G456D | testosterone | small molecule | agonist | DrugBank |
| *AR* | 367 | X | 66766355 | 66766355 | Missense_Mutation | *AR*:NM_000044:exon1:c.G1367A:p.G456D | nilutamide | small molecule | antagonist | DrugBank |
| *AR* | 367 | X | 66766355 | 66766355 | Missense_Mutation | *AR*:NM_000044:exon1:c.G1367A:p.G456D | fludrocortisone | small molecule | agonist | DrugBank |
| *AR* | 367 | X | 66766355 | 66766355 | Missense_Mutation | *AR*:NM_000044:exon1:c.G1367A:p.G456D | drostanolone | small molecule | agonist | DrugBank |
| *AR* | 367 | X | 66766355 | 66766355 | Missense_Mutation | *AR*:NM_000044:exon1:c.G1367A:p.G456D | nandrolone phenpropionate | small molecule | agonist | DrugBank |
| *AR* | 367 | X | 66766355 | 66766355 | Missense_Mutation | *AR*:NM_000044:exon1:c.G1367A:p.G456D | bicalutamide | small molecule | antagonist | DrugBank |
| *AR* | 367 | X | 66766355 | 66766355 | Missense_Mutation | *AR*:NM_000044:exon1:c.G1367A:p.G456D | fluoxymesterone | small molecule | agonist | DrugBank |
| *AR* | 367 | X | 66766355 | 66766355 | Missense_Mutation | *AR*:NM_000044:exon1:c.G1367A:p.G456D | drospirenone | small molecule | antagonist | DrugBank |
| *AR* | 367 | X | 66766355 | 66766355 | Missense_Mutation | *AR*:NM_000044:exon1:c.G1367A:p.G456D | danazol | small molecule | agonist | DrugBank |
| *AR* | 367 | X | 66766355 | 66766355 | Missense_Mutation | *AR*:NM_000044:exon1:c.G1367A:p.G456D | testosterone propionate | small molecule | agonist | DrugBank |
| *AR* | 367 | X | 66766355 | 66766355 | Missense_Mutation | *AR*:NM_000044:exon1:c.G1367A:p.G456D | boldenone | small molecule | agonist | DrugBank |
| *AR* | 367 | X | 66766355 | 66766355 | Missense_Mutation | *AR*:NM_000044:exon1:c.G1367A:p.G456D | calusterone | small molecule | N/A | DrugBank |
| *AR* | 367 | X | 66766355 | 66766355 | Missense_Mutation | *AR*:NM_000044:exon1:c.G1367A:p.G456D | flufenamic acid | small molecule | N/A | DrugBank |
| *AR* | 367 | X | 66766355 | 66766355 | Missense_Mutation | *AR*:NM_000044:exon1:c.G1367A:p.G456D | dihydrotestosterone | small molecule | N/A | DrugBank |
| *AR* | 367 | X | 66766355 | 66766355 | Missense_Mutation | *AR*:NM_000044:exon1:c.G1367A:p.G456D | methyltrienolone | small molecule | N/A | DrugBank |
| *AR* | 367 | X | 66766355 | 66766355 | Missense_Mutation | *AR*:NM_000044:exon1:c.G1367A:p.G456D | cyproterone | small molecule | antagonist | DrugBank |
| *AR* | 367 | X | 66766355 | 66766355 | Missense_Mutation | *AR*:NM_000044:exon1:c.G1367A:p.G456D | methyltestosterone | small molecule | agonist | DrugBank |
| *AR* | 367 | X | 66766355 | 66766355 | Missense_Mutation | *AR*:NM_000044:exon1:c.G1367A:p.G456D | nandrolone decanoate | small molecule | agonist | DrugBank |
| *AR* | 367 | X | 66766355 | 66766355 | Missense_Mutation | *AR*:NM_000044:exon1:c.G1367A:p.G456D | androgen receptor modulators and antagonists | - | - | MycancerGenome |
| *AR* | 367 | X | 66766355 | 66766355 | Missense_Mutation | *AR*:NM_000044:exon1:c.G1367A:p.G456D | pi3k inhibitors | - | - | MycancerGenome |
| *ATP8A1* | 10396 | 4 | 42602495 | 42602495 | Frame_Shift_Del | *ATP8A1*:NM_001105529:exon6:c.449delA:p.K150Rfs*2 | phosphatidylserine | small molecule | N/A | DrugBank |
| *CASP1* | 834 | 11 | 104905021 | 104905021 | Missense_Mutation | *CASP1*:NM_001223:exon2:c.C188T:p.P63L | minocycline | small molecule | negative modulator | DrugBank |
| *CSNK1G3* | 1456 | 5 | 122950080 | 122950080 | Missense_Mutation | *CSNK1G3*:NM_001364148:exon9:c.G899C:p.R300P | purvalanol a | small molecule | N/A | DrugBank |
| *DDR1* | 780 | 6 | 30860904 | 30860904 | Missense_Mutation | *DDR1*:NM_001202521:exon8:c.C1159T:p.P387S | imatinib | small molecule | antagonist | DrugBank |
| *DEK* | 7913 | 6 | 18264096 | 18264096 | Missense_Mutation | *DEK*:NM_001134709:exon2:c.G123C:p.E41D | flt3 inhibitors | - | - | MycancerGenome |
| *DEK* | 7913 | 6 | 18264096 | 18264096 | Missense_Mutation | *DEK*:NM_001134709:exon2:c.G123C:p.E41D | mek inhibitors | - | - | MycancerGenome |
| *DEK* | 7913 | 6 | 18264096 | 18264096 | Missense_Mutation | *DEK*:NM_001134709:exon2:c.G123C:p.E41D | jak2 inhibitors | - | - | MycancerGenome |
| *DEK* | 7913 | 6 | 18264096 | 18264096 | Missense_Mutation | *DEK*:NM_001134709:exon2:c.G123C:p.E41D | dot1l inhibitors | - | - | MycancerGenome |
| *GABRQ* | 55879 | X | 151821122 | 151821122 | Missense_Mutation | *GABRQ*:NM_018558:exon9:c.T1277C:p.L426P | lorazepam | small molecule | potentiator | DrugBank |
| *GABRQ* | 55879 | X | 151821122 | 151821122 | Missense_Mutation | *GABRQ*:NM_018558:exon9:c.T1277C:p.L426P | temazepam | small molecule | potentiator | DrugBank |
| *GABRQ* | 55879 | X | 151821122 | 151821122 | Missense_Mutation | *GABRQ*:NM_018558:exon9:c.T1277C:p.L426P | clobazam | small molecule | potentiator | DrugBank |
| *GABRQ* | 55879 | X | 151821122 | 151821122 | Missense_Mutation | *GABRQ*:NM_018558:exon9:c.T1277C:p.L426P | alprazolam | small molecule | potentiator | DrugBank |
| *GABRQ* | 55879 | X | 151821122 | 151821122 | Missense_Mutation | *GABRQ*:NM_018558:exon9:c.T1277C:p.L426P | chlordiazepoxide | small molecule | potentiator | DrugBank |
| *GABRQ* | 55879 | X | 151821122 | 151821122 | Missense_Mutation | *GABRQ*:NM_018558:exon9:c.T1277C:p.L426P | clorazepate | small molecule | potentiator | DrugBank |
| *GABRQ* | 55879 | X | 151821122 | 151821122 | Missense_Mutation | *GABRQ*:NM_018558:exon9:c.T1277C:p.L426P | midazolam | small molecule | potentiator | DrugBank |
| *GABRQ* | 55879 | X | 151821122 | 151821122 | Missense_Mutation | *GABRQ*:NM_018558:exon9:c.T1277C:p.L426P | flurazepam | small molecule | potentiator | DrugBank |
| *GABRQ* | 55879 | X | 151821122 | 151821122 | Missense_Mutation | *GABRQ*:NM_018558:exon9:c.T1277C:p.L426P | diazepam | small molecule | potentiator | DrugBank |
| *GABRQ* | 55879 | X | 151821122 | 151821122 | Missense_Mutation | *GABRQ*:NM_018558:exon9:c.T1277C:p.L426P | oxazepam | small molecule | potentiator | DrugBank |
| *GABRQ* | 55879 | X | 151821122 | 151821122 | Missense_Mutation | *GABRQ*:NM_018558:exon9:c.T1277C:p.L426P | triazolam | small molecule | potentiator | DrugBank |
| *GABRQ* | 55879 | X | 151821122 | 151821122 | Missense_Mutation | *GABRQ*:NM_018558:exon9:c.T1277C:p.L426P | clonazepam | small molecule | potentiator | DrugBank |
| *GABRQ* | 55879 | X | 151821122 | 151821122 | Missense_Mutation | *GABRQ*:NM_018558:exon9:c.T1277C:p.L426P | bromazepam | small molecule | potentiator | DrugBank |
| *GABRQ* | 55879 | X | 151821122 | 151821122 | Missense_Mutation | *GABRQ*:NM_018558:exon9:c.T1277C:p.L426P | nitrazepam | small molecule | potentiator | DrugBank |
| *GABRQ* | 55879 | X | 151821122 | 151821122 | Missense_Mutation | *GABRQ*:NM_018558:exon9:c.T1277C:p.L426P | venlafaxine | Efficacy | - | PharmGKB |
| *HCAR2* | 338442 | 12 | 123187239 | 123187239 | Missense_Mutation | *HCAR2*:NM_177551:exon1:c.T592C:p.F198L | niacin | small molecule | agonist | DrugBank |
| *IGSF10* | 285313 | 3 | 151161408 | 151161408 | Missense_Mutation | *IGSF10*:NM_001385061:exon6:c.C5327T:p.T1776I | pyridoxal phosphate | small molecule | cofactor | DrugBank |
| *MMP14* | 4323 | 14 | 23313927 | 23313927 | Missense_Mutation | *MMP14*:NM_004995:exon8:c.C1239A:p.D413E | marimastat | small molecule | inhibitor | DrugBank |
| *MTAP* | 4507 | 9 | 21818081 | 21818081 | Missense_Mutation | *MTAP*:NM_002451:exon4:c.C227A:p.A76E | adenine | small molecule | N/A | DrugBank |
| *MTAP* | 4507 | 9 | 21818081 | 21818081 | Missense_Mutation | *MTAP*:NM_002451:exon4:c.C227A:p.A76E | formycin | small molecule | N/A | DrugBank |
| *NDUFS6* | 4726 | 5 | 1816016 | 1816016 | Nonsense_Mutation | *NDUFS6*:NM_004553:exon4:c.C361T:p.Q121X | nadh | small molecule | N/A | DrugBank |
| *PPP1R13L* | 10848 | 19 | 45900258 | 45900258 | Frame_Shift_Del | *PPP1R13L*:NM_001142502:exon4:c.256delC:p.R86Gfs*138 | platinum compounds | Efficacy | - | PharmGKB |
| *PRKCB* | 5579 | 16 | 23999906 | 23999906 | Missense_Mutation | *PRKCB*:NM_002738:exon3:c.T283G:p.S95A | vitamin e | small molecule | N/A | DrugBank |
| *PRKCB* | 5579 | 16 | 23999906 | 23999906 | Missense_Mutation | *PRKCB*:NM_002738:exon3:c.T283G:p.S95A | dexmedetomidine | Other | - | PharmGKB |
| *PRKCQ* | 5588 | 10 | 6553041 | 6553041 | Frame_Shift_Del | *PRKCQ*:NM_001242413:exon3:c.233delA:p.N78Tfs*30 | staurosporine | small molecule | N/A | DrugBank |
| *PRKCQ* | 5588 | 10 | 6553041 | 6553041 | Frame_Shift_Del | *PRKCQ*:NM_001242413:exon3:c.233delA:p.N78Tfs*30 | phosphonothreonine | small molecule | N/A | DrugBank |
| *PRKCQ* | 5588 | 10 | 6553041 | 6553041 | Frame_Shift_Del | *PRKCQ*:NM_001242413:exon3:c.233delA:p.N78Tfs*30 | phosphonoserine | small molecule | N/A | DrugBank |
| *RUNX1T1* | 862 | 8 | 93026809 | 93026809 | Missense_Mutation | *RUNX1T1*:NM_175636:exon2:c.G355A:p.V119M | flt3 inhibitors | - | - | MycancerGenome |
| *RUNX1T1* | 862 | 8 | 93026809 | 93026809 | Missense_Mutation | *RUNX1T1*:NM_175636:exon2:c.G355A:p.V119M | mek inhibitors | - | - | MycancerGenome |
| *RUNX1T1* | 862 | 8 | 93026809 | 93026809 | Missense_Mutation | *RUNX1T1*:NM_175636:exon2:c.G355A:p.V119M | jak2 inhibitors | - | - | MycancerGenome |
| *RUNX1T1* | 862 | 8 | 93026809 | 93026809 | Missense_Mutation | *RUNX1T1*:NM_175636:exon2:c.G355A:p.V119M | dot1l inhibitors | - | - | MycancerGenome |

**Figure S1.** Flow chart of the selection process for renal anastomosing hemangioma patients.


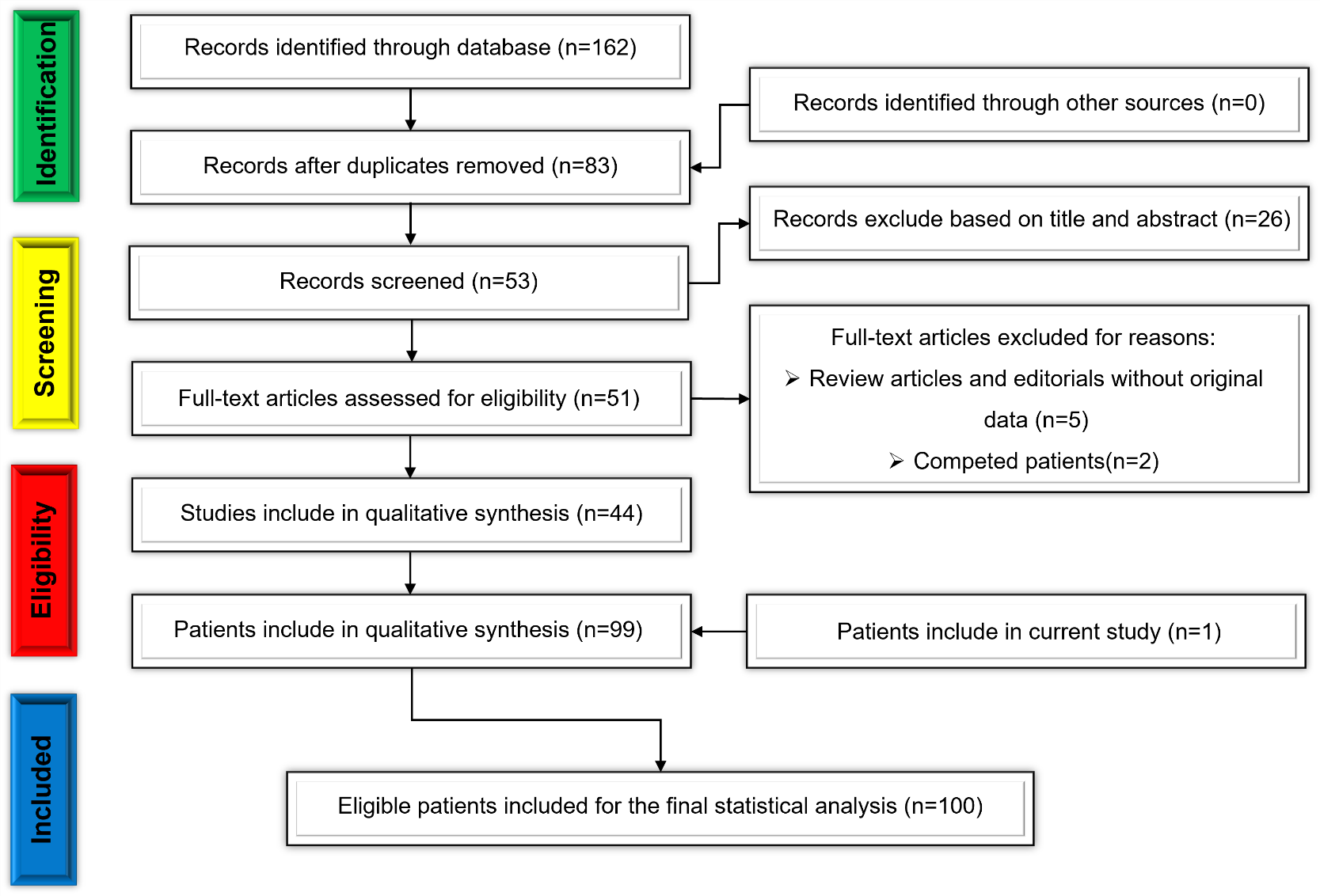

Supplement: Supplementary file 1 [file DataSheet1.docx]
